# Measurement Properties of Existing Patient-Reported Outcome Measures on Medication Adherence: Systematic Review

**DOI:** 10.2196/19179

**Published:** 2020-10-09

**Authors:** Yu Heng Kwan, Si Dun Weng, Dionne Hui Fang Loh, Jie Kie Phang, Livia Jia Yi Oo, Dan V Blalock, Eng Hui Chew, Kai Zhen Yap, Corrinne Yong Koon Tan, Sungwon Yoon, Warren Fong, Truls Østbye, Lian Leng Low, Hayden Barry Bosworth, Julian Thumboo

**Affiliations:** 1 Program in Health Services and Systems Research, Duke-NUS Medical School Singapore Singapore; 2 Department of Pharmacy National University of Singapore Singapore Singapore; 3 PULSES Centre Grant SingHealth Regional Health System Singapore Singapore; 4 Department of Rheumatology and Immunology Singapore General Hospital Singapore Singapore; 5 Center of Innovation to Accelerate Discovery and Practice Transformation (ADAPT) Durham Veterans Affairs Health Care System Durham, NC United States; 6 Department of Psychiatry and Behavioral Sciences Duke University School of Medicine Durham, NC United States; 7 Pharmacy Transformation Office National Healthcare Group Pharmacy Singapore Singapore; 8 Duke-NUS Medical School Singapore Singapore; 9 NUS Yong Loo Lin School of Medicine National University of Singapore Singapore Singapore; 10 Department of Family Medicine and Continuing Care Singapore General Hospital Singapore Singapore; 11 Post Acute and Continuing Care Outram Community Hospital Singapore Singapore; 12 Department of Population Health Sciences Duke University Medical Center Durham, NC United States; 13 School of Nursing Duke University Medical Center Durham, NC United States

**Keywords:** systematic review, reliability and validity, medication adherence, patient reported outcome measures

## Abstract

**Background:**

Medication adherence is essential for improving the health outcomes of patients. Various patient-reported outcome measures (PROMs) have been developed to measure medication adherence in patients. However, no study has summarized the psychometric properties of these PROMs to guide selection for use in clinical practice or research.

**Objective:**

This study aims to evaluate the quality of the PROMs used to measure medication adherence.

**Methods:**

This study was guided by the PRISMA (Preferred Reporting Items for Systematic Review and Meta-Analysis) guidelines. Relevant articles were retrieved from the EMBASE, PubMed, Cochrane Library, Web of Science, and CINAHL (Cumulative Index to Nursing and Allied Health Literature) databases. The PROMs were then evaluated based on the COnsensus-based Standards for the selection of health Measurement Instruments (COSMIN) guidelines.

**Results:**

A total of 121 unique medication adherence PROMs from 214 studies were identified. *Hypotheses testing for construct validity* and *internal consistency* were the most frequently assessed measurement properties. PROMs with at least a *moderate* level of evidence for ≥5 measurement properties include the Adherence Starts with Knowledge 20, Compliance Questionnaire-Rheumatology, General Medication Adherence Scale, Hill-Bone Scale, Immunosuppressant Therapy Barrier Scale, Medication Adherence Reasons Scale (MAR-Scale) revised, 5-item Medication Adherence Rating Scale (MARS-5), 9-item MARS (MARS-9), 4-item Morisky Medication Adherence Scale (MMAS-4), 8-item MMAS (MMAS-8), Self-efficacy for Appropriate Medication Adherence Scale, Satisfaction with Iron Chelation Therapy, Test of Adherence to Inhalers, and questionnaire by Voils. The MAR-Scale revised, MMAS-4, and MMAS-8 have been administered electronically.

**Conclusions:**

This study identified 121 PROMs for medication adherence and provided synthesized evidence for the measurement properties of these PROMs. The findings from this study may assist clinicians and researchers in selecting suitable PROMs to assess medication adherence.

## Introduction

Medication adherence is known as “the degree to which the person’s behaviour corresponds with the agreed recommendations from a healthcare provider” [1]. Medication adherence is essential when it comes to improving the health outcomes of patients, especially for chronic diseases [2,3]. However, only approximately half of the patients worldwide adhere to their prescribed medication regimen [4]. Reasons for medication nonadherence include complexity of the treatment regimen, poor communication with health care providers, and concerns about side effects from taking medications [5]. Poor medication adherence may lead to worse health outcomes and higher rates of mortality and morbidity [1,6]. Nonadherence also incurs a high cost burden to the health care system by increasing hospital visits as well as causing unnecessary escalation to more expensive treatments [1]. Therefore, improving medication adherence is key to improving treatment outcomes [7,8].

To successfully improve medication adherence, there is a need for the accurate assessment of medication adherence. Current practices for measuring medication include direct measures such as drug assays of blood or urine as well as indirect measures of adherence such as pill count, electronic monitoring devices, and the use of big data such as review of prescription records and claims [9,10]. Some of these measures are not time efficient and are likely to be unsustainable in clinical practice. Various patient-reported outcome measures (PROMs) such as the Morisky Medication Adherence Scale (MMAS) [11], Hill-Bone Compliance Scale [12], and Medication Adherence Rating Scale (MARS) [13] have been developed to measure self-reported adherence to medications. These PROMs may be useful in clinical practice because they are easy to administer. On the basis of the patients’ PROM ratings, health care professionals may be able to provide timely feedback. Thus, underlying issues that contribute to medication noncompliance can be addressed at the point of care [14].

A number of previous studies have been conducted to validate PROMs on medication adherence [12,15-17], and a previous systematic review found 14 PROMs that assessed adherence to inhaled asthma maintenance medication alone [18]. However, to date, there is no comprehensive review that summarizes the psychometric properties of PROMs for medication adherence, which is essential to guide the selection of suitable PROMs to evaluate medication adherence in patients. Hence, we carried out a systematic review to identify studies that investigated PROMs for medication adherence and to evaluate the quality of these PROMs.

## Methods

This study was conducted with reference to the PRISMA (Preferred Reporting Items for Systematic Review and Meta-Analysis) statement [19]. The measurement properties of each PROM were evaluated based on the COnsensus-based Standards for the selection of health Measurement Instruments(COSMIN) guidelines [20,21]. The COSMIN guidelines evaluate PROM development and the following 9 measurement properties: “content validity,” “structural validity,” “internal consistency,” “cross-cultural validity/measurement invariance,” test-retest “reliability,” “measurement error,” “criterion validity,” “hypotheses testing for construct validity,” and “responsiveness” [21,22]. Of note, the assessment of “convergent validity,” “discriminant validity,” and “known-group validity” falls under “hypotheses testing for construct validity” [23,24]. We also assumed that “concurrent validity” and “predictive validity” can be evaluated by the same measurement property, “hypothesis testing for construct validity,” and sensitivity to change can be evaluated under “responsiveness” as well [22,25,26].

### Search Strategy

The EMBASE, PubMed, Cochrane Library, Web of Science, and CINAHL databases were searched for relevant studies published before November 1, 2019. A search strategy (Multimedia Appendix 1) consisting of adherence, PROMs, and measurement properties was used. Search filters created by Terwee et al [27], which consists of a combination of search terms, were also used to enhance the sensitivity of searches, where available. For the adherence construct, synonyms such as compliance were used in the search. Duplicates were removed from the final search library.

### Study Selection

Articles included in this study were full-text publications in English, validating medication adherence PROMs, with the assessment of at least one measurement property listed in the COSMIN [24,28]. We excluded animal studies, case studies, narrative reviews, expert opinions, conference abstracts, and PROMs completed by proxy [29]. In total, 2 independent reviewers (SW and LO) screened the titles and abstracts of the studies according to the inclusion and exclusion criteria. Opinions from a third reviewer (YK) were sought in the event of any disagreements. The remaining full-text articles were then evaluated by the same 2 independent reviewers for inclusion and exclusion. Additional articles for inclusion into the final pool of articles were also identified from the reference list of articles during the full-text screening phase [30].

### Extraction of Data

The following data were then extracted from the included articles by 2 independent reviewers (SW and LO), where available:

- General characteristics of the study population: age, sample size, gender, and country.
- Characteristics of disease or condition: disease studied, duration of illness, or treatment.
- PROM characteristics: methods of administration, availability of electronic administration, language, response scale, domains, and number of items.

### Assessment of Methodological Quality

The methodological quality of the studies was assessed by 2 independent reviewers (SW and LO). Any disagreement was resolved in consultation with a third reviewer (JP). Each measurement property was assessed based on a 4-point scale: “inadequate,” “doubtful,” “adequate,” or “very good” [20,22]. The item with the worst rating under each measurement property would determine the overall rating for the specific measurement property [31].

On the basis of the COSMIN guidelines, it is recommended for the review team to determine before assessing the methodological quality of studies which outcome measurement instrument can be considered a reasonable gold standard [32]. The study team decided that there is currently no gold standard in the field of patient-reported outcomes that measure medication adherence [33,34]; thus, the assessment of criterion validity of the PROMs was not performed except when an abridged PROM was compared with the original long version, which will be regarded as the gold standard. This is in line with the consensus from the COSMIN panel that no gold standard exists for PROMs [35].

### Assessment of Psychometric Quality

The psychometric quality for each medication adherence instrument was assessed using the quality criteria by Terwee et al [36]. For each of the measurement properties evaluated in the included studies, a “positive (+),” “indeterminate (?),” or “negative (−)” rating was assigned based on the psychometric results.For example, for the internal consistency measurement property, the rating will be “+” if “at least low evidence for sufficient structural validity AND Cronbach’s alpha(s) ≥ 0.70 for each unidimensional scale or subscale.” The rating will be “−” if there is “at least low evidence for sufficient structural validity AND Cronbach’s alpha(s) < 0.70 for each unidimensional scale or subscale.” The rating will be “?” if the “criteria for at least low evidence for sufficient structural validity is not met” [36].

### Evidence Synthesis

For each PROM, an evidence synthesis across studies was conducted. First, we determined whether each measurement property for a PROM had overall “sufficient (+),” “insufficient (−),” “inconsistent (±),” or “indeterminate (?)” evidence for each measurement property of the PROM. For PROMs that were assessed in more than one study, the overall rating of the level of evidence for the PROM would be sufficient (+), indeterminate (?), or insufficient (–) if the individual studies were all consistently rated as positive (+), indeterminate (?), or negative (-), respectively. If the results of individual studies were inconsistent, the overall rating of the level of evidence for the PROM would be inconsistent.

We also graded the quality of evidence for each measurement property of PROM as “high,” “moderate,” “low,” or “very low” level of evidence based on the guidelines from the modified Grading of Recommendations Assessment, Development and Evaluation approach for systematic reviews of clinical trials [22,37].

## Results

### Search Results and Study Characteristics

**Figure 1:**
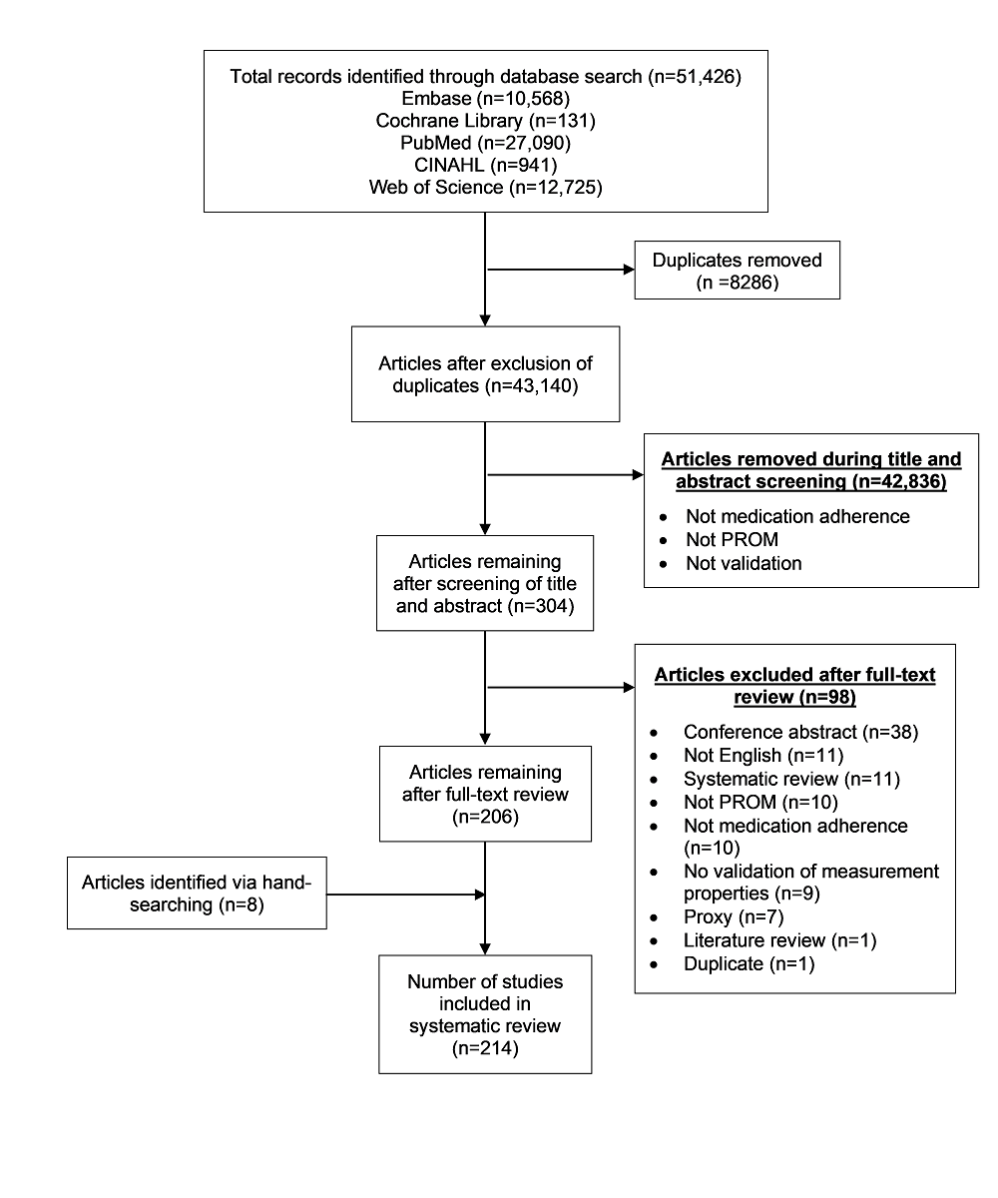
Preferred Reporting Items for Systematic Reviews and Meta-Analyses flow diagram for the systematic review. CINAHL: Cumulative Index to Nursing and Allied Health Literature; PROM: patient-reported outcome measure.

In total, 51,426 articles were retrieved from the 5 databases. After removing 8286 duplicates and 42,836 articles during title and abstract screening, 304 articles remained for full-text review. A total of 98 articles were further eliminated during full-text article screening. An additional 8 relevant articles were identified through hand-searching of the reference lists from the included articles to obtain a final list of 214 articles (Figure 1). A total of 240 PROMs were evaluated across 214 studies, and 121 unique medication adherence PROMs in 32 languages from 48 countries were identified (Table 1).

**Table 1:** Study characteristics of included articles (N=214).

Study characteristics		Values, n (%)
**Sample size^a^**		
	<50	17 (7.9)
	50-99	31 (14.4)
	100-199	62 (29.0)
	200-299	32 (15.0)
	300-399	23 (10.7)
	400-499	15 (7.0)
	>500	35 (16.4)
**Mean age (years)^a,b^**		
	<20	9 (4.2)
	20-39	28 (13.1)
	40-59	97 (45.3)
	60-79	55 (25.7)
	>80	2 (0.9)
**Proportion of males** **(%)** ^a^		
	0-20	15 (7.0)
	21-40	47 (22.0)
	41-60	78 (36.4)
	61-80	38 (17.8)
	81-100	23 (10.7)
**Mean treatment/disease duration^a^ (years)**		
	<1	3 (1.4)
	1-5	11 (5.1)
	6-10	27 (12.6)
	11-15	16 (7.5)
	>15	2 (0.9)

^a^Some values were not reported.

^b^Some values were reported as median, range, or not reported.

### PROM Characteristics

The characteristics of the medication adherence PROM are presented in Multimedia Appendix 2 [38-51]. The most extensively studied PROMs were the 8-item MMAS (MMAS-8)and 4-item MMAS(MMAS-4), which were studied in 27 and 18 studies, respectively. Among the 121 PROMs, 15 PROMs have been administered electronically—Adult AIDS Clinical Trials Group (AACTG) adherence items [52], Fredericksen et al [38], Item Response Theory-30 [53], LeastAbsolute Shrinkage and Selection Operator-10 [53], Medication Adherence Reasons Scale (MAR-Scale) [54], MAR-Scale (revised) [55], MARS [56], Medication Intake Survey-Asthma [57], MMAS-4 [54], MMAS-8 [58], Multiple Sclerosis Treatment Adherence Questionnaire [59], Probabilistic Medication Adherence Scale [56], Self-Rating Scale Item [52], visual analog scale [52], and WedAd-Q Questionnaire [60].

### Assessment of Methodological and Psychometric Quality

Outcomes on the assessment of methodological quality and study quality of PROMs are summarized in Multimedia Appendix 3 [13,17,38-54,56,57,59,61-248]. In terms of validity, “hypotheses testing for construct validity,”“structural validity,” and “content validity” were assessed in 181, 92, and 92 studies, respectively. In terms of reliability, “internal consistency” and “reliability” were assessed in 138 and 27 studies, respectively.

No studies assessed “measurement error” or “cross-cultural validity/measurement invariance.”Of note, 46 studies performed translation of PROMs, but none of them assessed measurement invariance or differential item functioning. Furthermore, although 75 studies assessed *PROM development,* only one of them obtained *very good* methodological quality.

### Evidence Synthesis

The results for the evidence synthesis for the PROMs are summarized in Table 2. PROMs with at least a *moderate* level of evidence for ≥5 measurement properties include the Adherence Starts with Knowledge 20 (ASK-20), Compliance Questionnaire-Rheumatology (CQR), General Medication Adherence Scale (GMAS), Hill-Bone Scale, Immunosuppressant Therapy Barrier Scale (ITBS), MAR-Scale revised, MARS-5, MARS-9, MMAS-4, MMAS-8, Self-efficacy for Appropriate Medication Adherence Scale (SEAMS), Satisfaction with Iron Chelation Therapy (SICT), Test of Adherence to Inhalers (TAI), and the questionnaire by Voils.

**Table 2:** Evidence synthesis of measurement properties for each patient-reported outcome measure.

PROM^a^	Number of studies	PROM development	Content validity	Structural validity	Internal consistency	Cross-cultural validity/measurement invariance	Reliability	Measurement error	Criterion validity	Hypotheses testing for construct validity	Responsiveness
Adult Asthma Adherence Questionnaire	1	0^b^	?^c^/B^d^	?/B	−^e^/C^f^	0	0	0	0	−/B	+^g^/B
Adult AIDS Clinical Trials Group	8	0	?/D^h^	?/B	+/A^i^	0	0	0	0	±^j^/C	+/C
Antidepressant Adherence Scale	1	D	?/D	0	−/D	0	0	0	0	−/C	0
Adherence Barrier Questionnaire	2	D	0	?/B	+/A	0	0	0	0	−/B	0
Adherence tool for chronic myelomonocytic leukemia	1	D	0	0	−/C	0	0	0	0	−/D	−/D
Adherence Evaluation of Osteoporosis Treatment Questionnaire-12	1	0	0	0	0	0	0	0	0	+/A	+/A
Antipsychotic Medication Beliefs and Attitudes Scale	1	D	+/C	−/B	+/A	0	0	0	0	+/B	0
Adherence to Pulmonary Rehabilitation Questionnaire	1	C	+/C	?/B	+/D	0	0	0	0	0	0
ARMS^k^	1	C	+/C	?/B	+/A	0	?/C	0	0	+/B	0
ARMS-7	1	0	?/C	−/A	+/A	0	+/B	0	0	0	0
ASK^l^-12	1	0	0	?/B	+/A	0	+/B	0	0	+/A	0
ASK-20	3	B	+/B	?/B	+/D	0	+/B	0	0	±/A	−/B
Attitudes to mesalamine questionnaire	1	D	0	0	0	0	0	0	0	+/A	+/A
Adherence self-report questionnaire	1	0	+/C	0	0	0	−/D	0	0	0	−/B
Axelsson et al^m^ [39]	1	D	0	?/B	+/A	0	0	0	0	+/C	0
Basel Assessment of Adherence to Immunosuppressive Medications Scale	1	0	+/B	?/B	+/D	—^n^	+/A	0	0	+/B	0
Brief Evaluation of Medication Influences and Beliefs	1	D	?/D	?/C	−/D	0	?/D	0	0	−/D	+/C
Beliefs Related to Medication Adherence	1	D	?/D	?/D	+/D	0	0	0	0	+/D	0
Brief Medication Adherence Scale	1	D	0	?/B	?/D	0	+/B	0	0	+/C	0
Beliefs about Medication Compliance Scale	1	D	+/C	?/B	+/A	0	0	0	0	0	0
Brief Medication Questionnaire	4	D	?/C	0	0	0	+/B	0	0	+/B	+/C
Center for Adherence Support Evaluation Adherence Index	2	0	0	0	0	0^n^	?/D	0	0	+/B	−/B
Chronic Disease Compliance Instrument	3	A	+/A	?/B	+/B	—^n^	0	0	0	−/B	0
CEAT-VIH	1	0	+/B	0	+/D	0	0	0	0	+/C	0
Chaiyachatiet al^m^ [40]	1	0	0	0	0	0	0	0	0	0	−/C
Compliance assessment	1	0	0	0	?/D	0	0	0	0	+/B	0
Cohort Study of Medication Adherence Among Older Adults self-report tool	1	D	0	?/D	0	0	0	0	0	+/B	+/B
CQR^o^	4	B	+/B	?/B	+/A	0^n^	±/B	0	0	±/B	+/A
CQR-5	1	0	+/A	+/D	+/A	0	0	0	0	+/B	−/B
Da et al^m^ [41]	1	0	0	0	0	0	0	0	0	−/C	−/B
DAI^p^	1	D	0	0	+/D	0	?/D	0	0	+/B	0
DAI-10	2	0	0	0	+/C	0	0	0	0	±/A	−/B
DAI-9	1	0	0	?/C	0	0	−/D	0	0	−/D	0
Diagnostic Adherence to Medication Scale	1	B	+/B	0	0	0	0	0	0	+/A	0
Demirtas et al^m^ [42]	1	B	+/B	?/A	+/A	0	?/C	0	0	+/C	0
Danish version of Medication Adherence Report Scale-4	1	0	?/D	0	+/C	0	0	0	0	−/D	0
Diabetes Management Questionnaire	1	D	?/C	0	+/A	0	−/C	0	0	+/B	0
Diabetes Medication Self-efficacy Scale	2	D	+/B	?/B	+/D	0^n^	+/B	0	0	+/C	−/B
Environmental Barriers to Adherence Scale	1	0	0	0	+/A	0	?/D	0	0	+/D	0
Eye-Drop Satisfaction Questionnaire	2	D	+/C	?/B	+/A	0	0	0	0	?/C	0
End-Stage Renal Disease Adherence Questionnaire	2	D	+/B	0	0	0^n^	+/C	0	0	−/B	0
Every Visit Adherence Questionnaire	1	0	0	0	0	0	0	0	0	+/D	0
Five-dimension adherence model	1	D	0	0	0	0	0	0	0	0	+/A
Fredericksen et al^m^ [249]	1	C	+/A	0	0	0	+/A	0	0	0	0
General adherence tendency measure	1	D	0	0	0	0	0	0	0	−/B	−/B
General Medicine Adherence Scale	3	C	+/B	+/A	+/A	0^n^	±/B	0	0	+/B	+/C
Godin et al^m^ [43]	2	C	?/C	0	0	0	0	0	0	−/C	+/C
GTCAT^q^	1	D	?/B	?/D	−/B	0	?/D	0	0	+/B	0
GTCAT (reduced)	2	0	?/B	−/C	−/A	0	+/B	0	0	−/B	0
Hill-Bone Scale	5	B	+/A	?/B	+/B	0^n^	0	0	0	+/A	0
Hill-Bone Scale (modified)	1	0	?/C	0	−/D	0^n^	0	0	0	0	0
HIV Intention Measure	1	C	+/B	?/B	+/A	0	0	0	0	+/C	0
HIV Symptom Quality of Life Adherence Questionnaire	1	D	0	?/B	+/C	0	0	0	0	+/B	0
Iraqi Anti-Diabetic Medication Adherence Scale	1	C	?/C	0	+/B	0^n^	?/C	0	0	+/B	+/C
Item Response Theory-30	1	B	+/B	?/B	0	0	0	0	0	?/C	+/B
Immunosuppressant Therapy Adherence Scale	2	0	0	?/B	+/D	0^n^	0	0	0	±/B	0
Immunosuppressant Therapy Barrier Scale	1	C	+/B	?/B	+/A	0	0	0	0	+/A	+/A
Kennedy et al^m^ [44]	1	D	+/B	0	0	0	0	0	0	0	0
Kerr et al^m^ [45]	1	D	0	0	0	0	0	0	0	−/D	−/D
Least absolute shrinkage and selection operator-10	1	B	+/B	?/B	0	0	0	0	0	?/C	+/B
Long-Term Medication Behaviour Self-Efficacy Scale	1	D	?/D	0	0	0	0	0	0	0	0
Modified Drug Adherence Work-up Tool	2	0	?/D	?/D	+/D	0	0	0	0	−/B	+/B
Medication Adherence Questionnaire	1	D	?/D	0	+/D	0^n^	+/D	0	0	0	0
MAR-Scale^r^	1	C	?/D	?/B	−/A	0	−/D	0	0	+/D	0
MAR-Scale (revised)	2	B	+/B	?/B	+/A	0	0	0	0	+/B	0
MARS^s^	8	D	+/D	?/B	±/A	0^n^	?/D	0	0	±/B	±/B
MARS-10	1	0	?/D	?/B	+/A	0^n^	0	0	0	+/B	+/B
MARS-5	8	0	+/B	?/B	+/B	0^n^	±/B	0	0	±/A	±/B
MARS-9	2	0	+/B	?/B	+/A	0	−/B	0	0	±/B	0
MASES^t^	2	D	+/A	?/B	+/A	0	−/D	0	0	±/A	0
MASES-R	1	0	0	?/B	+/D	0	−/C	0	0	−/B	0
Medication Adherence Self-Report Inventory	6	D	0	0	+/B	0	+/D	0	0	+/A	+/A
Medication adherence scale	1	D	+/C	−/A	+/A	0	0	0	0	−/B	0
Medication adherence survey	1	B	+/B	0	0	0	0	0	0	+/D	0
Medication Adherence Estimation and Differentiation Scale	1	C	?/B	+/A	+/A	0	0	0	0	+/D	−/A
Medication Intake Survey-Asthma	1	B	+/B	0	0	0	−/B	0	0	+/B	0
MMAS^u^-4	18	0	+/C	?/B	−/B	0	±/B	0	0	±/A	−/B
MMAS-7	1	0	0	0	0	0^n^	0	0	0	+/B	0
MMAS-8	27	0	+/B	±/A	±/A	0^n^	+/B	0	0	±/A	−/B
MMAS-9	1	0	0	+/A	−/A	0	?/C	0	0	−/B	0
Medication Nonpersistence Scale	1	C	?/C	+/A	+/D	0	0	0	0	+/C	−/D
Medical Outcomes Study General Adherence Scale	1	0	0	0	+/D	0	0	0	0	−/C	0
Multiple Sclerosis Treatment Adherence Questionnaire	1	C	?/B	0	−/A	0	0	0	0	+/A	0
Outcome Expectations for Osteoporosis Medication Adherence Scale	2	D	+/B	±/A	+/A	0	0	0	0	−/C	0
Perceived Barriers to Antiretroviral Therapy Adherence Scale	1	B	+/C	?/B	?/C	0^n^	−/C	0	0	+/C	0
Pictographic Self-Efficacy Scale	1	D	+/B	0	−/A	0	−/D	0	0	−/B	0
Patient Rating of Compliance Scale	1	0	0	0	0	0	0	0	0	+/B	0
Patient Preference Questionnaire	1	D	+/D	0	+/D	0	0	0	0	0	0
Probabilistic Medication Adherence Scale	1	C	±/C	−/A	+/C	0	0	0	0	0	0
Number of pills taken or prescribed	2	0	0	0	?/D	0	−/D	0	0	±/C	0
Questionnaire for Adherence with Topical Treatments in Psoriasis	1	D	+/B	0	0	0	0	0	0	±/D	0
Question of Interest	1	0	?/D	0	0	0	0	0	0	−/D	−/D
SCI^v^	2	0	?/C	+/A	0	0	−/B	0	0	±/A	−/B
SCI-R	1	0	+/B	?/B	+/A	0^n^	?/D	0	0	+/D	+/D
Strathclyde Compliance Risk Assessment Tool	2	0	0	0	+/B	0	0	0	0	+/B	+/B
Summary of Diabetes Self-care Activities	2	0	0	0	0	0	0	0	0	±/C	0
Self-Efficacy for Appropriate Medication Adherence Scale	3	B	+/B	?/B	+/A	0	+/A	0	0	±/A	0
Self-efficacy scale	1	D	?/B	?/B	+/A	0	0	0	0	?/D	0
Self-report measures of adherence	1	0	0	0	0	0	?/B	0	0	+/B	0
Self-report on adherence	1	0	0	0	0	0	0	0	0	−/C	+/C
Self-Efficacy for Osteoporosis Medication Adherence Scale	2	D	+/B	−/A	+/A	0	0	0	0	+/C	0
Self-Reported Adherence Questionnaire	1	0	D	0	0	0	0	0	0	?/D	0
Satisfaction with Iron Chelation Therapy	1	B	+/B	?/B	+/A	0	0	0	0	+/A	0
Sidorkiewicz et al^m^ [46]	1	B	+/B	0	0	0	0	0	0	+/C	0
Simplified Medication Adherence Questionnaire	2	D	?/C	0	+/C	0	+/A	0	0	+/B	−/A
Stages of Change Model Questionnaire	2	D	0	0	0	0	0	0	0	+/C	0
Special Projects of National Significance Adherence Survey	1	0	0	0	+/D	0	0	0	0	+/C	0
Self-Rating Scale Item	4	0	0	0	0	0	0	0	0	+/C	+/C
Test of Adherence to Inhalers	1	B	?/B	?/B	+/D	0	+/A	0	0	−/C	−/A
Tan et al [47]	1	C	?/C	?/B	+/A	0	+/A	0	0	+/B	0
Treatment Adherence Survey-Patient Version	1	D	0	0	0	0	+/B	0	0	−/C	0
Therapeutic Adherence Scale for Hypertensive Patients	1	0	0	+/A	+/A	0	0	0	0	+/B	−/B
Topical Therapy Adherence Questionnaire	1	D	+/D	0	+/D	0	0	0	0	0	0
Turcu-știolică et al^m^ [48]	1	D	+/D	0	−/D	0	0	0	0	−/D	0
Visual analog scale	4	0	0	0	0	0	−/D	0	0	+/B	0
Validated Hemophilia Regimen Treatment Adherence Scale—On-Demand	1	D	+/D	0	+/B	0	+/B	0	0	±/B	0
Validated Hemophilia Regimen Treatment Regimen Treatment Adherence Scale-Prophylaxis	1	D	+/D	0	+/C	0	+/C	0	0	−/C	0
Voils et al^m^ [49]	4	C	+/B	+/A	+/A	0^n^	−/A	0	0	±/A	−/B
Vreeman et al^m^ [50]	2	C	?/C	0	0	0^n^	0	0	0	−/C	−/C
Web-Ad-Q Questionnaire	1	C	+/C	0	0	0	+/D	0	0	+/C	0
Wilson et al^m^ [51]	3	B	+/B	0	+/A	0	0	0	0	+/D	−/A

^a^PROM: patient-reported outcome measurement.

^b^0: Measurement properties were not assessed by the study.

^c^?: intermediate.

^d^B: moderate.

^e^−: insufficient.

^f^C: low.

^g^+: sufficient.

^h^D: very low.

^i^A: high.

^j^±: inconsistent.

^k^ARMS: Adherence to Refills and Medications Scale.

^l^ASK: Adherence Starts with Knowledge questionnaire.

^m^PROMs without proper names are labeled based on the last name of the first author who developed the instrument.

^n^Only translation was done. Cross-cultural validation was not the aim of the study.

^o^CQR: Compliance Questionnaire on Rheumatology.

^p^DAI: Drug Attitude Inventory.

^q^GTCAT: Glaucoma Treatment Compliance Assessment Tool.

^r^MAR-Scale: Medication Adherence Reasons Scale.

^s^MARS: Medication Adherence Rating Scale.

^t^MASES: Medication Adherence Self-efficacy Scale.

^u^MMAS: Morisky Medication Adherence Scale.

^v^SCI: Self-Care Inventory.

## Discussion

To the best of our knowledge, this is the first systematic review that comprehensively summarized PROMs for medication adherence based on the COSMIN guidelines [21,22]. Among the 214 included articles, we identified 121 unique PROMs for medication adherence. Our study revealed the most commonly evaluated medication adherence PROMs to be the MMAS-8, MMAS-4, AACTG, MARS, and MARS-5. However, being more commonly evaluated does not mean that these PROMs have the best psychometric properties. Moreover, based on the number of studies for each PROM in Table 2, most of the PROMs have too few studies to provide a strong evidence base for their use. 

Among the 15 PROMs that have been administered electronically, 3 PROMs (MAR-Scale revised, MMAS-4, and MMAS-8) have at least a *moderate* level of evidence for ≥5 measurement properties. Electronic administration of PROMs to measure medication adherence may be appealing in health care settings, as it may reduce the administrative burden for data collection and data entry. In addition, as web-based interventions to improve medication adherence become increasingly commonplace [250], electronic PROMs may be incorporated into web-based platforms to assess the effectiveness of these web-based interventions.

Despite a few studies claiming the use of certain PROMs and objective measures as the *gold standard* for measuring medication adherence, we deliberately omitted evaluating *criterion validity* for these studies. As mentioned in the introduction, although objective measures such as pill count, electronic monitoring devices, and big data may measure adherence indirectly, these measures are laborious, costly, and sometimes invasive, making them unsuitable for routine clinical use. Furthermore, all these surrogate measures, including PROMs, do not predict any real biological outcomes such as a reduction in viral load, blood pressure, or glucose concentration in determining medication adherence in patients. Hence, none of these measures can be deemed as a *gold standard* [10].

Measurement error was not evaluated because none of the studies reported the standard error of measurement, smallest detectable change, or limits of agreements required by the COSMIN. In addition, although translations of PROMs were performed in 46 studies, none of these studies assessed measurement invariance or differential item functioning; therefore, cross-cultural validity was not evaluated for any of the PROMs in this study. Moreover, only one study examined the interpretability of PROMs in the form of minimal detectable change for the MMAS-8 [251]. Further study on measurement error, cross-cultural validity, and interpretability of medication adherence PROMs is warranted.

The strengths of this study include using COSMIN guidelines, which are well regarded as a consensus-based standard for evaluating the measurement properties of PROMs [23]. The COSMIN Risk of Bias checklist employed in this study is an improvement from the original COSMIN checklist with several improvements in the standards for evaluation [21,22]. We also used sensitive search filters to retrieve and include as many potentially relevant articles as possible.

One limitation related to this study was that the selection of articles and evaluation of psychometric properties were subjective in nature and may have been prone to judgment bias. Nevertheless, the requirement by COSMIN to have 2 independent reviewers and the need for a third reviewer to reach a consensus in the case of any discrepancy occurring has helped reduce the risk of judgment bias [22,252].

### Conclusions

In summary, 121 unique medication adherence PROMs were identified in 214 studies. On the basis of the COSMIN guidelines, PROMs with at least a *moderate* level of evidence for ≥5 measurement properties include the ASK-20, CQR, GMAS, Hill-Bone Scale, ITBS, MAR-Scale revised, MARS-5, MARS-9, MMAS-4, MMAS-8, SEAMS, SICT, TAI, and questionnaire by Voils. Of these, only the GMAS has *sufficient (+)* ratings for at least four measurement properties. We believe this study would assist clinicians and researchers in selecting suitable PROMs to measure medication adherence among patients. Future research may consider validating measurement errors and cross-cultural validity to further improve the insights on the measurement properties of these PROMs.

## Appendix

Multimedia Appendix 1 Search strategy.

Multimedia Appendix 2 Characteristics of patient reported outcome measures.

Multimedia Appendix 3 Assessment of psychometric properties of patient-reported outcome measures.

## Abbreviations

AACTG: Adult AIDS Clinical Trials Group
ASK-20: Adherence Starts with Knowledge 20
COSMIN: Consensus-based Standards for the selection of health Measurement INstruments
CQR: Compliance Questionnaire-Rheumatology
GMAS: General Medication Adherence Scale
ITBS: Immunosuppressant Therapy Barrier Scale
MAR-Scale: Medication Adherence Reasons Scale
MARS: Medication Adherence Rating Scale
MARS-5: five-item Medication Adherence Rating Scale
MARS-9: nine-item Medication Adherence Rating Scale
MMAS-4: four-item Morisky Medication Adherence Scale
MMAS-8: eight-item Morisky Medication Adherence Scale
PROM: patient-reported outcome measure
PULSES: Population-based, Unified, Learning System for Enhances and Sustainable Health
SEAMS: self-efficacy for appropriate medication adherence scale
SICT: satisfaction with iron chelation therapy
TAI: Test of Adherence to Inhalers
