# Supplementary material for: Measurement Properties of Existing Patient-Reported Outcome Measures on Medication Adherence: Systematic Review
Source: J Med Internet Res. 2020 Oct 9;22(10):e19179. doi: 10.2196/19179 (PMC7584986; doi:10.2196/19179)
Supplement: Multimedia Appendix 1 [file jmir_v22i10e19179_app1.docx]

**Supplementary Table 1: Search strategy**

| **Database** | **EMBASE** | **CINAHL** | **COCHRANE Library** | **PubMed** | **Web of Science** |
| --- | --- | --- | --- | --- | --- |
| Date restriction |  |  | Between Jan 1900 and Oct 2019 |  |  |
| Construct: adherence | \| 1 \| ('patient compliance'/de OR 'medication compliance'/exp) \| \| --- \| --- \| \| 2 \| persisten* \| \| 3 \| ("Patient compliance":ti,ab OR "User compliance":ti,ab OR "patient adherence":ti,ab OR "treatment adherence":ti,ab OR "Patient adherence":ti,ab OR "Patient cooperation":ti,ab OR "Patient non adherence":ti,ab OR "Patient non compliance":ti,ab OR "Patient nonadherence":ti,ab OR "Patient non-adherence":ti,ab OR "Patient noncompliance":ti,ab OR "Patient non-compliance":ti,ab OR "adherence to therapy":ti,ab OR "adherence to treatment":ti,ab OR "Family planning clinic attendance":ti,ab OR "compliance to therapy":ti,ab OR "compliance to treatment":ti,ab OR "therapy adherence":ti,ab OR "therapy compliance":ti,ab OR "treatment adherence":ti,ab OR "treatment compliance":ti,ab OR "dosage adherence":ti,ab OR "dosage compliance":ti,ab OR "dose adherence":ti,ab OR "dose compliance":ti,ab OR "dosing adherence":ti,ab OR "dosing compliance":ti,ab OR "drug adherence":ti,ab OR "drug compliance":ti,ab OR "drug intake compliance":ti,ab OR "drug regimen adherence":ti,ab OR "drug regimen compliance":ti,ab OR "medication adherence":ti,ab OR "medication intake adherence":ti,ab OR "adherence to pharmacotherapy":ti,ab OR "compliance with pharmacotherapy") \| \| 4 \| #1 OR #2 OR #3 \| | \| 1 \| persisten* \| \| --- \| --- \| \| 2 \| (MH "Patient Compliance+") OR (MH "Medication Compliance") OR (MH "Patient Compliance+") OR (MH "Compliance with Therapeutic Regimen (Saba CCC)") OR (MH "Compliance with Medication Regimen (Saba CCC)") OR (MH "Compliance Behavior (Iowa NOC)") \| \| 3 \| (TI "Patient compliance" OR TI "User compliance" OR TI "patient adherence" OR TI "treatment adherence" OR TI "Patient adherence" OR TI "Patient cooperation" OR TI "Patient non adherence" OR TI "Patient non compliance" OR TI "Patient nonadherence" OR TI "Patient non-adherence"OR TI "Family planning clinic attendance"OR TI "Patient noncompliance" OR TI "Patient non-compliance" OR TI "adherence to therapy" OR TI "adherence to treatment" OR TI "compliance to therapy" OR TI "compliance to treatment" OR TI "therapy adherence" OR TI "therapy compliance" OR TI "treatment adherence" OR TI "treatment compliance" OR TI "dosage adherence" OR TI "dosage compliance" OR TI "dose adherence" OR TI "dose compliance" OR TI "dosing adherence" OR TI "dosing compliance" OR TI "drug adherence" OR TI "drug compliance" OR TI "drug intake compliance" OR TI "drug regimen adherence" OR TI "drug regimen compliance" OR TI "medication adherence" OR TI "medication intake adherence" OR TI "adherence to pharmacotherap*" OR TI "compliance with pharmacotherap*") \| \| 4 \| (AB "Patient compliance" OR AB "User compliance" OR AB "patient adherence" OR AB "treatment adherence" OR AB "Patient adherence" OR AB "Patient cooperation" OR AB "Patient non adherence" OR AB "Patient non compliance" OR AB "Patient nonadherence" OR AB "Patient non-adherence"OR AB "Family planning clinic attendance"OR AB "Patient noncompliance" OR AB "Patient non-compliance" OR AB "adherence to therapy" OR AB "adherence to treatment" OR AB "compliance to therapy" OR AB "compliance to treatment" OR AB "therapy adherence" OR AB "therapy compliance" OR AB "treatment adherence" OR AB "treatment compliance" OR AB "dosage adherence" OR AB "dosage compliance" OR AB "dose adherence" OR AB "dose compliance" OR AB "dosing adherence" OR AB "dosing compliance" OR AB "drug adherence" OR AB "drug compliance" OR AB "drug intake compliance" OR AB "drug regimen adherence" OR AB "drug regimen compliance" OR AB "medication adherence" OR AB "medication intake adherence" OR AB "adherence to pharmacotherap*" OR AB "compliance with pharmacotherap*") \| \| 5 \| #1 OR #2 OR #3 OR #4 \| | \| 1 \| persisten* \| \| --- \| --- \| \| 2 \| ("Patient Compliance"):kw \| \| 3 \| ("Patient compliance" OR "User compliance" OR "patient adherence" OR "treatment adherence" OR "Patient adherence" OR "Patient cooperation" OR "Patient non adherence" OR "Patient non compliance" OR "Patient nonadherence" OR "Patient non-adherence" OR "Family planning clinic attendance" OR "Patient noncompliance" OR "Patient non-compliance" OR "adherence to therapy" OR "adherence to treatment" OR "compliance to therapy" OR "compliance to treatment" OR "therapy adherence" OR "therapy compliance" OR "treatment adherence" OR "treatment compliance" OR "dosage adherence" OR "dosage compliance" OR "dose adherence" OR "dose compliance" OR "dosing adherence" OR "dosing compliance" OR "drug adherence" OR "drug compliance" OR "drug intake compliance" OR "drug regimen adherence" OR "drug regimen compliance" OR "medication adherence" OR "medication intake adherence" OR "adherence to pharmacotherap*" OR "compliance with pharmacotherap*") \| \| 4 \| #1 OR #2 OR #3 \| | \| 1 \| medication adheren* \| \| --- \| --- \| \| 2 \| patient complian* OR patient compliance[MeSH] \| \| 3 \| persisten* \| \| 4 \| #1 OR #2 OR #3 \| | \| 1 \| TS=(persisten*) \| \| --- \| --- \| \| 2 \| TS=("Patient compliance" OR "User compliance" OR "patient adherence" OR "treatment adherence" OR "Patient adherence" OR "Patient cooperation" OR "Patient non adherence" OR "Patient non compliance" OR "Patient nonadherence" OR "Patient non-adherence" OR "Patient noncompliance" OR "adherence to therap*" OR "adherence to treatment*" OR "Family planning clinic attendance" OR "compliance to therap*" OR "compliance with treatment*" OR "therapy adherence" OR "therapy compliance" OR "treatment adherence" OR "treatment compliance" OR "dos* adherence" OR "dos* compliance" OR "drug adherence" OR "drug compliance" OR "drug intake compliance" OR "drug regimen adherence" OR "drug regimen compliance" OR "medication adherence" OR "medication intake adherence" OR "adherence to pharmacotherap*" OR "compliance with pharmacotherap*") \| \| 3 \| #1 OR #2 \| |
| Population: general patients | \| 5 \| ('patient reported'/exp) \| \| --- \| --- \| \| 6 \| (patient reported*:ti,ab OR "PROM":ti,ab) \| \| 7 \| #5 OR #6 \| |  |  |  |  |
| Construct: PROMs |  | \| 6 \| (MH "patient reported*") \| \| --- \| --- \| \| 7 \| (TI patient reported*) \| \| 8 \| (AB patient reported*) \| \| 9 \| #6 OR #7 OR #8 \| | \| 5 \| (patient reported*):kw \| \| --- \| --- \| |  |  |
| Instrument: PRO | \| 8 \| instrument*:ti,ab OR scale*:ti,ab OR questionnaire*:ti,ab OR measure*:ti OR methods:ti OR outcome-measurement*:ti,ab OR (test:ti,ab OR tests:ti,ab) OR "outcomes research"/de OR "treatment outcome"/de OR "psychologic test"/de OR "measurement"/de OR "functional assessment"/de OR "pain assessment"/de OR "questionnaire"/de OR "rating scale"/de \| \| --- \| --- \| | \| 10 \| (AB instrument* OR TI instrument*) OR (AB scale* OR TI scale*) OR (AB questionnaire* OR TI questionnaire*) OR (AB measure* OR TI measure*) OR (TI methods) OR (AB outcome-measurement* OR TI outcome-measurement*) OR ((AB test OR TI test) OR (AB tests OR TI tests)) \| \| --- \| --- \| \| 11 \| (MM "Outcomes Research") OR (MH "Treatment Outcomes+") OR (MH "Psychological Tests+") OR (AB "measurement") OR (MH "Functional Assessment") OR (MH "Pain Measurement") OR (MH "Questionnaires+") OR (MH "Scales") \| \| 12 \| #10 OR #11 \| | \| 6 \| (Questionnaires OR interview):kw \| \| --- \| --- \| \| 7 \| (instrument* OR scale* OR Questionnaire* OR measure* OR methods OR outcome measurement* OR (test OR tests)) \| \| 8 \| #6 OR #7 \| | \| 5 \| patient reported* \| \| --- \| --- \| \| 6 \| (HR-PRO[tiab] OR HRPRO[tiab] OR((patient[tiab] OR self[tiab] OR proxy[tiab]) AND ((report[tiab] OR reported[tiab] OR reporting[tiab]) OR (rated[tiab] OR rating[tiab] OR ratings[tiab]) OR based[tiab] OR (assessed[tiab] OR assessment[tiab] OR assessments[tiab]))) OR ((subjective[tiab] AND (index[tiab] OR indices[tiab] OR instrument[tiab] OR instruments[tiab] OR measure[tiab] OR measures[tiab] OR questionnaire[tiab] OR questionnaires[tiab] OR profile[tiab] OR profiles[tiab] OR scale[tiab] OR scales[tiab] OR score[tiab] OR scores[tiab] OR status[tiab] OR survey[tiab] OR surveys[tiab])))) \| \| 7 \| #5 OR #6 \| |  |
| Evaluation set: Measurement properties | \| 9 \| 'intermethod comparison'/exp OR 'data collection method'/exp OR 'validation study'/exp OR 'feasibility study'/exp OR 'pilot study'/exp OR 'psychometry'/exp OR 'reproducibility'/exp OR reproducib*:ab,ti OR "audit":ab,ti OR psychometr*:ab,ti OR clinimetr*:ab,ti OR clinometr*:ab,ti OR "observer variation"/exp OR "observer variation":ab,ti OR "discriminant analysis"/exp OR "validity"/exp OR reliab*:ab,ti OR valid*:ab,ti OR "coefficient":ab,ti OR "internal consistency":ab,ti OR (cronbach*:ab,ti AND ("alpha":ab,ti OR "alphas":ab,ti)) OR "item correlation":ab,ti OR "item correlations":ab,ti OR "item selection":ab,ti OR "item selections":ab,ti OR "item reduction":ab,ti OR "item reductions":ab,ti OR "agreement":ab,ti OR "precision":ab,ti OR "imprecision":ab,ti OR "precise values":ab,ti OR "test–retest":ab,ti OR ("test":ab,ti AND "retest":ab,ti) OR (reliab*:ab,ti AND ("test":ab,ti OR "retest":ab,ti)) OR "stability":ab,ti OR "interrater":ab,ti OR "inter-rater":ab,ti OR "intrarater":ab,ti OR "intra-rater":ab,ti OR "intertester":ab,ti OR "inter-tester":ab,ti OR "intratester":ab,ti OR "intra-tester":ab,ti OR "interobeserver":ab,ti OR "inter-observer":ab,ti OR "intraobserver":ab,ti OR "intra-observer":ab,ti OR "intertechnician":ab,ti OR "intertechnician":ab,ti OR "intratechnician":ab,ti OR "intra-technician":ab,ti OR "interexaminer":ab,ti OR "inter-examiner":ab,ti OR "intraexaminer":ab,ti OR "intraexaminer":ab,ti OR "interassay":ab,ti OR "inter-assay":ab,ti OR "intraassay":ab,ti OR "intra-assay":ab,ti OR "interindividual":ab,ti OR "inter-individual":ab,ti OR "intraindividual":ab,ti OR "intra-individual":ab,ti OR "interparticipant":ab,ti OR "inter-participant":ab,ti OR "intraparticipant":ab,ti OR "intra-participant":ab,ti OR "kappa":ab,ti OR "kappas":ab,ti OR "coefficient of variation":ab,ti OR repeatab*:ab,ti OR (replicab*:ab,ti OR "repeated":ab,ti AND ("measure":ab,ti OR "measures":ab,ti OR "findings":ab,ti OR "result":ab,ti OR "results":ab,ti OR "test":ab,ti OR "tests":ab,ti)) OR generaliza*:ab,ti OR generalisa*:ab,ti OR "concordance":ab,ti OR ("intraclass":ab,ti AND correlation*:ab,ti) OR "discriminative":ab,ti OR "known group":ab,ti OR "factor analysis":ab,ti OR "factor analyses":ab,ti OR "factor structure":ab,ti OR "factor structures":ab,ti OR "dimensionality":ab,ti OR subscale*:ab,ti OR "multitrait scaling analysis":ab,ti OR "multitrait scaling analyses":ab,ti OR "item discriminant":ab,ti OR "interscale correlation":ab,ti OR "interscale correlations":ab,ti OR ("error":ab,ti OR "errors":ab,ti AND (measure*:ab,ti OR correlat*:ab,ti OR evaluat*:ab,ti OR "accuracy":ab,ti OR "accurate":ab,ti OR "precision":ab,ti OR "mean":ab,ti)) OR "individual variability":ab,ti OR "interval variability":ab,ti OR "rate variability":ab,ti OR "variability analysis":ab,ti OR ("uncertainty":ab,ti AND ("measurement":ab,ti OR "measuring":ab,ti)) OR "standard error of measurement":ab,ti OR sensitiv*:ab,ti OR responsive*:ab,ti OR ("limit":ab,ti AND "detection":ab,ti) OR "minimal detectable concentration":ab,ti OR interpretab*:ab,ti OR (small*:ab,ti AND ("real":ab,ti OR "detectable":ab,ti) AND ("change":ab,ti OR "difference":ab,ti)) OR "meaningful change":ab,ti OR "minimal important change":ab,ti OR "minimal important difference":ab,ti OR "minimally important change":ab,ti OR "minimally important difference":ab,ti OR "minimal detectable change":ab,ti OR "minimal detectable difference":ab,ti OR "minimally detectable change":ab,ti OR "minimally detectable difference":ab,ti OR "minimal real change":ab,ti OR "minimal real difference":ab,ti OR "minimally real change":ab,ti OR "minimally real difference":ab,ti OR "ceiling effect":ab,ti OR "floor effect":ab,ti OR "item response model":ab,ti OR "irt":ab,ti OR "rasch":ab,ti OR "differential item functioning":ab,ti OR "dif":ab,ti OR "computer adaptive testing":ab,ti OR "item bank":ab,ti OR "cross-cultural equivalence":ab,ti \| \| --- \| --- \| | \| 13 \| AB 'intermethod comparison' OR (MH "Data Collection Methods+") OR (MM "Validation Studies") OR 'feasibility study'/exp OR (MM "Pilot Studies")OR (MH "Measurement Issues and Assessments+") OR (MM "Reproducibility of Results") OR (MM "Discriminant Analysis") OR (MH "Validity+") \| \| --- \| --- \| \| 14 \| AB reproducib* OR AB "audit" OR AB psychometr* OR AB clinimetr* OR AB clinometr* OR AB "observer variation" OR AB reliab* OR AB valid* OR AB "coefficient" OR AB "internal consistency" OR (AB cronbach* AND (AB "alpha" OR AB "alphas")) OR AB "item correlation" OR AB "item correlations" OR AB "item selection" OR AB "item selections" OR AB "item reduction" OR AB "item reductions" OR AB "agreement" OR AB "precision" OR AB "imprecision" OR AB "precise values" OR AB "test–retest" OR (AB "test" AND AB "retest") OR (AB reliab* AND (AB "test" OR AB "retest")) OR AB "stability" OR AB "interrater" OR AB "inter-rater" OR AB "intrarater" OR AB "intra-rater" OR AB "intertester" OR AB "inter-tester" OR AB "intratester" OR AB "intra-tester" OR AB "interobeserver" OR AB "inter-observer" OR AB "intraobserver" OR AB "intra-observer" OR AB "intertechnician" OR AB "intertechnician" OR AB "intratechnician" OR AB "intra-technician" OR AB "interexaminer" OR AB "inter-examiner" OR AB "intraexaminer" OR AB "intraexaminer" OR AB "interassay" OR AB "inter-assay" OR AB "intraassay" OR AB "intra-assay": OR AB "interindividual" OR AB "inter-individual" OR AB "intraindividual" OR AB "intra-individual" OR AB "interparticipant" OR AB "inter-participant" OR AB "intraparticipant" OR AB "intra-participant" OR AB "kappa" OR AB "kappas" OR AB "coefficient of variation" OR AB repeatab* OR (AB replicab* OR AB "repeated" AND (AB "measure" OR AB "measures" OR AB "findings" OR AB "result" OR AB "results" OR AB "test" OR "tests")) OR AB generaliza* OR AB generalisa* OR AB "concordance" OR (AB "intraclass" AND AB correlation*) OR AB "discriminative" OR AB "known group" OR AB "factor analysis" OR AB "factor analyses" OR AB "factor structure" OR AB "factor structures" OR AB "dimensionality" OR AB subscale* OR AB "multitrait scaling analysis" OR AB "multitrait scaling analyses" OR AB "item discriminant" OR AB "interscale correlation" OR AB "interscale correlations" OR (AB "error" OR AB "errors" AND (AB measure* OR AB correlat* OR AB evaluat* OR AB "accuracy" OR AB "accurate" OR AB "precision" OR AB "mean")) OR AB "individual variability" OR AB "interval variability" OR AB "rate variability" OR AB "variability analysis" OR (AB "uncertainty" AND (AB "measurement" OR AB "measuring")) OR AB "standard error of measurement" OR AB sensitiv* OR AB responsive* OR (AB "limit" AND AB "detection") OR AB "minimal detectable concentration" OR AB interpretab* OR (AB small* AND (AB "real" OR AB "detectable") AND (AB "change" OR AB "difference")) OR AB "meaningful change" OR AB "minimal important change" OR AB "minimal important difference" OR AB "minimally important change" OR AB "minimally important difference" OR AB "minimal detectable change" OR AB "minimal detectable difference" OR AB "minimally detectable change" OR AB "minimally detectable difference" OR AB "minimal real change" OR AB "minimal real difference" OR AB "minimally real change" OR AB "minimally real difference" OR AB "ceiling effect" OR AB "floor effect" OR AB "item response model" OR AB "irt" OR "rasch" OR AB "differential item functioning" OR AB "dif" OR AB "computer adaptive testing" OR AB "item bank" OR AB "cross-cultural equivalence" \| \| 15 \| TI reproducib* OR TI "audit" OR TI psychometr* OR TI clinimetr* OR TI clinometr* OR TI "observer variation" OR TI reliTI* OR TI valid* OR TI "coefficient" OR TI "internal consistency" OR (TI cronbach* AND (TI "alpha" OR TI "alphas")) OR TI "item correlation" OR TI "item correlations" OR TI "item selection" OR TI "item selections" OR TI "item reduction" OR TI "item reductions" OR TI "agreement" OR TI "precision" OR TI "imprecision" OR TI "precise values" OR TI "test–retest" OR (TI "test" AND TI "retest") OR (TI reliTI* AND (TI "test" OR TI "retest")) OR TI "stTIility" OR TI "interrater" OR TI "inter-rater" OR TI "intrarater" OR TI "intra-rater" OR TI "intertester" OR TI "inter-tester" OR TI "intratester" OR TI "intra-tester" OR TI "interobeserver" OR TI "inter-observer" OR TI "intraobserver" OR TI "intra-observer" OR TI "intertechnician" OR TI "intertechnician" OR TI "intratechnician" OR TI "intra-technician" OR TI "interexaminer" OR TI "inter-examiner" OR TI "intraexaminer" OR TI "intraexaminer" OR TI "interassay" OR TI "inter-assay" OR TI "intraassay" OR TI "intra-assay": OR TI "interindividual" OR TI "inter-individual" OR TI "intraindividual" OR TI "intra-individual" OR TI "interparticipant" OR TI "inter-participant" OR TI "intraparticipant" OR TI "intra-participant" OR TI "kappa" OR TI "kappas" OR TI "coefficient of variation" OR TI repeatTI* OR (TI replicTI* OR TI "repeated" AND (TI "measure" OR TI "measures" OR TI "findings" OR TI "result" OR TI "results" OR TI "test" OR "tests")) OR TI generaliza* OR TI generalisa* OR TI "concordance" OR (TI "intraclass" AND TI correlation*) OR TI "discriminative" OR TI "known group" OR TI "factor analysis" OR TI "factor analyses" OR TI "factor structure" OR TI "factor structures" OR TI "dimensionality" OR TI subscale* OR TI "multitrait scaling analysis" OR TI "multitrait scaling analyses" OR TI "item discriminant" OR TI "interscale correlation" OR TI "interscale correlations" OR (TI "error" OR TI "errors" AND (TI measure* OR TI correlat* OR TI evaluat* OR TI "accuracy" OR TI "accurate" OR TI "precision" OR TI "mean")) OR TI "individual variTIility" OR TI "interval variTIility" OR TI "rate variTIility" OR TI "variTIility analysis" OR (TI "uncertainty" AND (TI "measurement" OR TI "measuring")) OR TI "standard error of measurement" OR TI sensitiv* OR TI responsive* OR (TI "limit" AND TI "detection") OR TI "minimal detectTIle concentration" OR TI interpretTI* OR (TI small* AND (TI "real" OR TI "detectTIle") AND (TI "change" OR TI "difference")) OR TI "meaningful change" OR TI "minimal important change" OR TI "minimal important difference" OR TI "minimally important change" OR TI "minimally important difference" OR TI "minimal detectTIle change" OR TI "minimal detectTIle difference" OR TI "minimally detectTIle change" OR TI "minimally detectTIle difference" OR TI "minimal real change" OR TI "minimal real difference" OR TI "minimally real change" OR TI "minimally real difference" OR TI "ceiling effect" OR TI "floor effect" OR TI "item response model" OR TI "irt" OR "rasch" OR TI "differential item functioning" OR TI "dif" OR TI "computer adaptive testing" OR TI "item bank" OR TI "cross-cultural equivalence" \| \| 16 \| #13 OR #14 OR #15 \| | \| 9 \| (methods OR "Validation Studies" OR "Comparative Study" OR "psychometrics" OR "outcome assessment (health care)" OR "observer variation" OR "Health Status Indicators" OR "reproducibility of results" OR "Discriminant Analysis"):kw \| \| --- \| --- \| \| 10 \| ("outcome measure*" OR psychometr* OR clinimetr* OR clinometr* OR "outcome assessment" OR "observer variation" OR reproducib* OR reliab* OR unreliab* OR valid* OR "coefficient of variation" OR coefficient OR homogeneity OR homogeneous OR "internal consistency" OR (cronbach* AND (alpha OR alphas)) OR (item AND (correlation* OR selection* OR reduction*)) OR agreement OR precision OR imprecision OR "precise values" OR test–retest OR (test AND retest) OR (reliab* AND (test OR retest)) OR stability OR interrater OR inter-rater OR intrarater OR intra-rater OR intertester OR inter-tester OR intratester OR intra-tester OR interobserver OR inter-observer OR intraobserver OR intra-observer OR intertechnician OR inter-technician OR intratechnician OR intra-technician OR interexaminer OR inter-examiner OR intraexaminer OR intra-examiner OR interassay OR inter-assay OR intraassay OR intra-assay OR interindividual OR inter-individual OR intraindividual OR intra-individual OR interparticipant OR inter-participant OR intraparticipant OR intra-participant OR kappa OR kappa’s OR kappas OR repeatab* OR interexaminer OR inter-examiner OR intraexaminer OR intra-examiner OR interassay OR inter-assay OR intraassay OR intra-assay OR interindividual OR inter-individual OR intraindividual OR intra-individual OR interparticipant OR inter-participant OR intraparticipant OR intra-participant OR kappa OR kappa’s OR kappas OR repeatab* OR ((replicab* OR repeated) AND (measure OR measures OR findings OR result OR results OR test OR tests)) OR generaliza* OR generalisa* OR concordance OR (intraclass AND correlation*) OR discriminative OR "known group" OR "factor analysis" OR "factor analyses" OR "factor structure" OR "factor structures" OR dimension* OR subscale* OR (multitrait AND scaling AND (analysis OR analyses)) OR interexaminer OR inter-examiner OR intraexaminer OR intra-examiner OR interassay OR inter-assay OR intraassay OR intra-assay OR interindividual OR inter-individual OR intraindividual OR intra-individual OR interparticipant OR inter-participant OR intraparticipant OR intra-participant OR kappa OR kappa’s OR kappas OR repeatab* OR ((replicab* OR repeated) AND (measure OR measures OR findings OR result OR results OR test OR tests)) OR generaliza* OR generalisa* OR concordance OR (intraclass AND correlation*) OR discriminative OR "known group" OR "factor analysis" OR "factor analyses" OR "factor structure" OR "factor structures" OR dimension* OR subscale* OR (multitrait AND scaling AND (analysis OR analyses)) OR "item discriminant" OR "interscale correlation*" OR error OR errors OR "individual variability" OR "interval variability" OR "rate variability" OR (variability AND (analysis OR values)) OR (uncertainty AND (measurement OR measuring)) OR "standard error of measurement" OR sensitiv* OR responsive* OR (limit AND detection) OR "minimal detectable concentration" OR interpretab* OR ((minimal OR minimally OR clinical OR clinically) AND (important OR significant OR detectable) AND (change OR difference)) OR (small* AND (real OR detectable) AND (change OR difference)) OR "meaningful change" OR "ceiling effect" OR "floor effect" OR "Item response model" OR IRT OR Rasch OR "Differential item functioning" OR DIF OR "computer adaptive testing" OR "item bank" OR "cross-cultural equivalence") \| \| 11 \| #9 OR #10 \| | \| 8 \| instrumentation[sh] OR methods[sh] OR Validation Studies OR Comparative Study OR “psychometrics”[MeSH] OR psychometr*[tiab] OR clinimetr*[tw] OR clinometr*[tw] OR “outcome assessment (health care)”[MeSH] OR outcome assessment[tiab] OR outcome measure*[tw] OR “Health Status Indicators”[Mesh] OR “reproducibility of results”[MeSH] OR reproducib*[tiab] OR “discriminant analysis”[MeSH] OR reliab*[tiab] OR unreliab*[tiab] OR valid*[tiab] OR coefficient[tiab] OR homogeneity[tiab] OR homogeneous[tiab] OR “internal consistency”[tiab] OR (cronbach*[tiab] AND (alpha[tiab] OR alphas[tiab])) OR (item[tiab] AND (correlation*[tiab] OR selection*[tiab] OR reduction*[tiab])) OR agreement[tiab] OR precision[tiab] OR imprecision[tiab] OR “precise values”[tiab] OR test–retest[tiab] OR (test[tiab] AND retest[tiab]) OR (reliab*[tiab] AND (test[tiab] OR retest[tiab])) OR stability[tiab] OR interrater[tiab] OR inter-rater[tiab] OR intrarater[tiab] OR intra-rater[tiab] OR interobserver[tiab] OR inter-observer[tiab] OR intraobserver[tiab] OR intra-observer[tiab] OR interindividual[tiab] OR inter-individual[tiab] OR intraindividual[tiab] OR intra-individual[tiab] OR interparticipant[tiab] OR inter-participant[tiab] OR intraparticipant[tiab] OR intra-participant[tiab] OR kappa[tiab] OR kappa’s[tiab] OR kappas[tiab] OR repeatab*[tiab] OR ((replicab*[tiab] OR repeated[tiab]) AND (measure[tiab] OR measures[tiab] OR findings[tiab] OR result[tiab] OR results[tiab] OR test[tiab] OR tests[tiab])) OR generaliza*[tiab] OR generalisa*[tiab] OR (intraclass[tiab] AND correlation*[tiab]) OR discriminative[tiab] OR “known group”[tiab] OR factor analysis[tiab] OR factor analyses[tiab] OR dimension*[tiab] OR subscale*[tiab] OR (multitrait[tiab] AND scaling[tiab] AND (analysis[tiab] OR analyses[tiab])) OR item discriminant[tiab] OR interscale correlation*[tiab] OR error[tiab] OR errors[tiab] OR “individual variability”[tiab] OR (variability[tiab] AND (analysis[tiab] OR values[tiab])) OR (uncertainty[tiab] AND (measurement[tiab] OR measuring[tiab])) OR “standard error of measurement”[tiab] OR sensitiv*[tiab] OR responsive*[tiab] OR ((minimal[tiab] OR minimally[tiab] OR clinical[tiab] OR clinically[tiab]) AND (important[tiab] OR significant[tiab] OR detectable[tiab]) AND (change[tiab] OR difference[tiab])) OR (small*[tiab] AND (real[tiab] OR detectable[tiab]) AND (change[tiab] OR difference[tiab])) OR meaningful change[tiab] OR “ceiling effect”[tiab] OR “floor effect”[tiab] OR “Item response model”[tiab] OR IRT[tiab] OR Rasch[tiab] OR “Differential item functioning”[tiab] OR DIF[tiab] OR “computer adaptive testing”[tiab] OR “item bank”[tiab] OR “cross-cultural equivalence”[tiab])) \| \| --- \| --- \| | \| 4 \| TS=(patient reported*) \| \| --- \| --- \| \| 5 \| TS=(instrument* OR scale* OR Questionnaire* OR measure* OR methods OR outcome measurement* OR (test OR tests) OR interview) \| \| 6 \| TS=(instrumentation OR methods OR "Validation Study" OR "Comparative Study" OR "psychometrics" OR psychometr* OR clinimetr* OR clinometr* OR "outcome assessment" OR "outcome measure*" OR "observer variation" OR "observer variation" OR "Health Status Indicators" OR "reproducibility of results" OR reproducib* OR "discriminant analysis" OR reliab* OR unreliab* OR valid* OR "coefficient of variation" OR coefficient OR homogeneity OR homogeneous OR "internal consistency" OR (cronbach* AND (alpha OR alphas)) OR (item AND (correlation* OR selection* OR reduction*)) OR agreement OR precision OR imprecision OR "precise values" OR test–retest OR (test AND retest) OR (reliab* AND (test OR retest)) OR stability OR interrater OR inter-rater OR intrarater OR intra-rater OR intertester OR inter-tester OR intratester OR intra-tester OR interobserver OR inter-observer OR intraobserver OR intra-observer OR intertechnician OR inter-technician OR intratechnician OR intra-technician OR interexaminer OR inter-examiner OR intraexaminer OR intra-examiner OR interassay OR inter-assay OR intraassay OR intra-assay OR interindividual OR inter-individual OR intraindividual OR intra-individual OR interparticipant OR inter-participant OR intraparticipant OR intra-participant OR kappa OR kappa’s OR kappas OR repeatab* OR interexaminer OR inter-examiner OR intraexaminer OR intra-examiner OR interassay OR inter-assay OR intraassay OR intra-assay OR interindividual OR inter-individual OR intraindividual OR intra-individual OR interparticipant OR inter-participant OR intraparticipant OR intra-participant OR kappa OR kappa’s OR kappas OR repeatab* OR ((replicab* OR repeated) AND (measure OR measures OR findings OR result OR results OR test OR tests)) OR generaliza* OR generalisa* OR concordance OR (intraclass AND correlation*) OR discriminative OR "known group" OR "factor analysis" OR "factor analyses" OR "factor structure" OR "factor structures" OR dimension* OR subscale* OR (multitrait AND scaling AND (analysis OR analyses)) OR interexaminer OR inter-examiner OR intraexaminer OR intra-examiner OR interassay OR inter-assay OR intraassay OR intra-assay OR interindividual OR inter-individual OR intraindividual OR intra-individual OR interparticipant OR inter-participant OR intraparticipant OR intra-participant OR kappa OR kappa’s OR kappas OR repeatab* OR ((replicab* OR repeated) AND (measure OR measures OR findings OR result OR results OR test OR tests)) OR generaliza* OR generalisa* OR concordance OR (intraclass AND correlation*) OR discriminative OR "known group" OR "factor analysis" OR "factor analyses" OR "factor structure" OR "factor structures" OR dimension* OR subscale* OR (multitrait AND scaling AND (analysis OR analyses)) OR "item discriminant" OR "interscale correlation*" OR error OR errors OR "individual variability" OR "interval variability" OR "rate variability" OR (variability AND (analysis OR values)) OR (uncertainty AND (measurement OR measuring)) OR "standard error of measurement" OR sensitiv* OR responsive* OR (limit AND detection) OR "minimal detectable concentration" OR interpretab* OR ((minimal OR minimally OR clinical OR clinically) AND (important OR significant OR detectable) AND (change OR difference)) OR (small* AND (real OR detectable) AND (change OR difference)) OR "meaningful change" OR "ceiling effect" OR "floor effect" OR "Item response model" OR IRT OR Rasch OR "Differential item functioning" OR DIF OR "computer adaptive testing" OR "item bank" OR "cross-cultural equivalence") \| |
| Final search strategy | \| 10 \| #4 AND #7 AND #8 AND #9 \| \| --- \| --- \| \| 11 \| #10 AND [1-1-1900]/sd NOT [1-11-2019]/sd \| | \| 17 \| #5 AND #9 AND #12 AND #16 \| \| --- \| --- \| \| 18 \| #17 (Until 31^st^ October 2019) \| | \| 12 \| #4 AND #5 AND #8 AND #11 \| \| --- \| --- \| | \| 9 \| #4 AND #7 AND #8 \| \| --- \| --- \| \| 10 \| #9 with date restriction (1900/01/01 to 2019/10/31) \| | \| 7 \| #3 AND #4 AND #5 AND #6 \| \| --- \| --- \| \| 8 \| #7 AND Last Two Weeks (backdate from 21^st^ November 2019) \| \| 9 \| #7 with date restriction (until 7^th^ Nov 2019) \| |

Reproduced from YH Kwan et al. " Development of an Item Bank to Measure the Extent of and Reasons for Medication Adherence" JMIR (In print)
