# Supplementary material for: Measurement Properties of Existing Patient-Reported Outcome Measures on Medication Adherence: Systematic Review
Source: J Med Internet Res. 2020 Oct 9;22(10):e19179. doi: 10.2196/19179 (PMC7584986; doi:10.2196/19179)
Supplement: Multimedia Appendix 2 [file jmir_v22i10e19179_app2.docx]

**Supplementary Table 2.** Characteristics of PROMs.

| **PROM** | **No. of studies** | **Languages** | **Countries** | **Conditions** | **Methods of administration** | **Electronic administration** | **Domains** | **No of domains (& items)** | **Response scale** |
| --- | --- | --- | --- | --- | --- | --- | --- | --- | --- |
| AAAQ | 1 | English | US | Asthma | Self-administered | No | General adherence; specific barriers | 1 (5) | 6 point Likert Scale |
| AACTG Adherence Questionnaire | 8 | English, Spanish | US, Tanzania | HIV (ART), T2DM, TB, Hepatitis C | Self-/Interviewer administered | Yes | Adherence | 1 (10) | Numerical, 5 point Likert, categorical, dichotomous, MCQ |
| AAS | 1 | English | Canada | Depression | Self-administered | No | Adherence | 1 (4) | Numerical rating scale |
| ABQ | 2 | German, English | Germany | AF, HIV (ART) | Self-administered | No | Adherence barriers | 1 (16) | 4 point Likert Scale |
| ADEOS-12 | 1 | French | France | Osteoporosis | Self-administered | No | Beliefs; perceptions; behaviour; information | 4 (12) | 3 and 5 point Likert Scale |
| AMBAS | 1 | Portuguese | Portugal | Antipsychotic | Self-administered | No | Barriers to adherence; positive beliefs about medications | 2 (12) | 5 point Likert Scale |
| APRQ | 1 | English | Malaysia | COPD | Self-administered | No | Disease management behaviour; perceived treatment benefits; emotional factors; perceived severity of disease; barriers towards treatment; coping attitude | 6 (18) | 5 point Likert Scale |
| ARMS | 1 | English | US | Chronic disease | Self-administered | No | Medication taking; prescription refill | 2 (12) | 4 point Likert Scale |
| ARMS-7 | 1 | Turkish | Turkey | Chronic disease | Self-administered | No | Adherence | 1 (7) | 4 point Likert Scale |
| ASK-12 | 1 | English | US | Asthma, T2DM, CHF | Self-administered | No | Behaviour; health beliefs; inconvenience/forgetfulness | 3 (12) | ? |
| ASK-20 | 3 | English, Japanese | US, Japan | Asthma, COPD, depression, CHF, T2DM | Self-administered | No | Adherence barriers | 1 (20) | 5 point Likert Scale |
| ASRQ | 1 | English | UK | HTN | Self-administered | No | Adherence | 1( 6) | 6 point Likert |
| Attitudes to mesalamine questionnaire | 1 | English | US | Ulcerative colitis | Self-administered | No | Adherence barriers | 1 (10) | ? |
| *Axelsson et al*^a^ | 1 | ? | Sweden | Asthma | Self-administered | No | Medication routines; self-adjusting the medication; concerns about side-effects | 3 (10) | 5 point Likert Scale |
| BAASIS | 1 | Portuguese | Brazil | Kidney transplant | Self-administered | No | Drug-taking NA, drug holidays, timing NA, dose reductions | 4 (4) | 6 point Likert Scale |
| BEMIB | 1 | English | US | Schizophrenia | Self-administered | No | Risk of illness; benefits, barriers & cost of treatment, cues to act | 5 (8) | 5 point Likert Scale |
| BERMA | 1 | English | US | Generic | Self-administered | No | Memory for medications; ability to deal with health professionals; attitudes about drugs | 3 (54) | 5 point Likert |
| BMAS | 1 | Chinese (Cantonese) | China (HK) | Schizophrenia/ Schizoaffective disorder | Self-administered | No | Medication adherence behaviors; attitudes towards taking medication | 2 (10) | Numeric rating scale |
| BMCS | 1 | English | US | Heart failure | Self-administered | No | Medication beliefs | 1 (12) | 5 point Likert |
| BMQ | 4 | English, Thai | US, Thailand | Chronic disease, HTN, TB | Self/Telephone administered | No | Regimen, belief, recall & access screen | 4 (13) | Yes/no, 3 point Likert Scale |
| CASE adherence index | 2 | Thai | Thailand | ART | Self-administered | No | Adherence | 1 (3) | Numerical |
| CDCI | 3 | English, Finnish | Australia, US, Finland | Asthma, diabetes, chronic diseases | Self-administered | No | Compliance; sense of normality; support from doctors; support from nurses; support from parents; experience of results; fear of complications; fear of asthma attacks/hypoglycaemia; support from friends; energy and will power; motivation | 11 (42) | 5 point Likert Scale |
| CEAT-VIH | 1 | Romanian | Romania | HIV | Interviewer-administered | No | Adherence behaviours; antecedents of non-adherence; treatment knowledge; doctor-patient relationship; perceived difficulty of adherence and side effects; information and use of strategies; outcome expectations; health status; self-efficacy; beliefs regarding adherence-related time | 10 (20) | 3 and 5 point Likert Scale, dichotomous |
| *Chaiyachati et al*^a^ | 1 | isiZulu | South Africa | HIV | Self-administered | No | Recall of missed dose (7 day), recall of late dose (7 day), adherence (1 month), 30 day VAS, last time of missed dose | 5 (5) | Recall, 6 point Likert Scale, VAS |
| Compliance assessment | 1 | ? | Finland | Neuroleptic treatment | Self-administered | No | Adherence | ? | 5 point Likert Scale |
| CoSMO self-report tool | 1 | English | US | HTN | Self-administered | No | Adherence | 1 (4) | 4 point Likert, categorical |
| CQR | 4 | English, Dutch, Korean, Turkish | South Korea, Netherlands, US, Turkey | Rheumatic diseases, Behcet's disease (vasculitis) | Self-administered | No | Regularity of medicine use; believe to medication benefit; interruption to medication; expectation about medicine; barriers | 5 (19) | 4 point Likert Scale |
| CQR5 | 1 | English | UK | Rheumatic diseases | Self-administered | No | Adherence | 1 (5) | 4 point Likert Scale |
| *Da et al*^a^ | 1 | Chinese | China | HIV | Self-administered | No | Adherence | 1 (4) | Number of days/doses |
| DAI | 1 | English | Canada | Schizophrenia | Self-administered | No | Adherence | 1 (30) | 7-point scale |
| DAI-10 | 2 | Polish | Poland | Acute psychotic crisis | Self-administered | No | Attitude towards compliance | 1 (10) | True/false |
| DAI-9 | 1 | Swedish | Sweden | Schizophrenia | Self-administered | No | Adherence | 1 (9) | 4 point Likert Scale |
| DAMS | 1 | English | UK | Generic | Interviewer-administered | No | Adherence | 1 (6) | ? |
| *Demirtas et al* ^a^ | 1 | Turkish |  | T2DM | Interviewer-administered questionnaire | No | Emotional difficulties in compliance; physical difficulties in compliance; changing difficulties of habits in compliance; acceptance difficulties in compliance; awareness difficulties in compliance; diet difficulties in compliance; denial difficulties in compliance | 7 (30) | 5 point Likert Scale |
| DMARS-4 | 1 | Danish | Turkey | Cancer pain | Self-administered | No | Adherence | 1 (4) | 5 point Likert Scale |
| DMQ | 1 | English | US | T1DM | Self-administered | No | Adherence | 1 (20) | 5 point Likert Scale |
| DMSS | 2 | English, Chinese | US, China | T2DM | Self-administered | No | Self-efficacy | 1 (19) | 3 point Likert Scale |
| EBAS | 1 | English | US | DM | Self-administered | No | Adherence behaviours | 1 (4) | Likert Scale |
| EDSQ | 2 | English, French | France | Glaucoma | Self-administered | No | Patient &treatment characteristics; patient-clinician relationship; patient experience& knowledge; patient-treatment interaction | 6 (43) | Continuous, dichotomous, categorical, ordinal |
| ESRD-AQ (PESRD-EQ) | 2 | Portuguese | Portugal | ESRD | Self-administered | No | General clinical information; dialysis attendance; medication use; fluid restrictions; diet | 5 (46) | Likert Scale, MCQ, binary |
| Every Visit Adherence Questionnaire | 1 | English | Australia | HIV ART | Self-administered | No | Adherence | 1 (1) | Open-ended |
| Five-dimension adherence model | 1 | Lithuanian | Lithuania | Bacterial infection | Self-administered | No | Healthcare-related, condition-related, therapy-related factors&patient-related factors | 4 (20) | Yes/no, 5 point Likert Scale |
| *Fredericksen et al*^a^ | 1 | English, Spanish | US | HIV | Computer-administered questionnaire | Yes | Adherence behaviours; adherence barriers | 2 (7) | Yes/no, check all that apply |
| General adherence tendency measure | 1 | French | Canada | T2DM | Self-administered | No | General tendency | 1 (1) | 5 point Likert Scale |
| GMAS | 3 | English, Urdu | Pakistan, Saudi Arabia | Chronic disease | Self-administered | No | Non-adherence due to patient’s behavior; non-adherence due to additional disease and pill burden; cost-related non-adherence | 3 (11) | ? |
| *Godin et al*^a^ | 1 | English, French | Canada | T2DM | Self-administered | No | Adherence | 1 (9) | Range |
| GTCAT | 1 | English | US | Glaucoma | Self-administered | No | Compliance | 1 (47) | 10 point Likert Scale |
| GTCAT (reduced) | 2 | Portuguese, English | Portugal, US | Glaucoma | Self-administered | No | Compliance | 1 (27) | 5 point Likert Scale |
| Hill-Bone Scale | 5 | English, German Portuguese, | US, Portugal, Germany | HTN | Self-administered | No | Reducing sodium intake; appointment keeping; medication taking | 3 (14) | 4 point Likert Scale |
| Hill-Bone Scale (modified) | 1 | Arabic | Saudi Arabia | HTN | ? | No | Frequency of behaviour related medication taking; appointment keeping | 2 (11) | 4 point Likert Scale |
| HIV-IM | 1 | English, Spanish | US | ART | Self-administered | No | Intention to adhere | 1 (53) | 6 point Likert Scale |
| HIV-SQUAD | 1 | French | France | ART | Self-administered | No | HRQoL; side-effects; adherence | 3 (26) | 5 point Likert Scale, yes/no, VAS |
| IADMAS | 1 | Arabic | Iraq | T2DM | Self-administered | No | Adherence | 1 (8) | 5 point Likert, yes/no |
| IRT-30 | 1 | English | US | ART | Self-administered | Yes | Adherence | 1 (30) | 5, 11 point Likert, yes/no |
| ITAS | 2 | Portuguese, English | Brazil, US | Liver transplant immunosuppression | Self-administered | No | Forgetfulness; carelessness; neglect; cessation due to feeling worse | 4 (4) | 4 point Likert Scale |
| ITBS | 1 | English | US | Organ transplant allograft rejection | Self- administered | No | Adherence barriers | 1 (13) | 5 point Likert Scale |
| *Kennedy et al*^a^ | 1 | English | US | ART | Self-administered | No | Adherence | 1 (26) | 5 point Likert Scale |
| *Kerr et al*^a^ | 1 | English | Canada | Injection drugs | Self-administered | No | Adherence | 1 (1) | 5 point Likert Scale |
| Lasso-10 | 1 | English | US | ART | Self-administered | Yes | Persistence | 1 (10) | 5, 6, 10 point Likert, yes/no |
| Long-Term Medication Behaviour Self-Efficacy Scale | 1 | English | US | Generic | Self-administered | No | Personal attributes, environmental factors, task related and behavioural factors | 3 (33) | VAS |
| M-DRAW | 2 | English |  | T2DM, Chronic disease | Self-administered | No | Adherence barriers | 1 (13) | 4 point Likert Scale |
| MAQ | 1 | Kannada, Malayalam | India | HTN | Self-administered | No | Adherence | 1 (8) | Yes/no |
| MAR-Scale | 1 | English | US | Asthma, dyslipidemia | Self-administered | Yes | Managing issues; belief issues; multiple medication issues; availability issues; forgetfulness issues | 5 (15) | 5 point Likert Scale |
| MAR-Scale (revised) | 2 | English | US | Asthma, dyslipidemia | Self-administered | Yes | Practical issues; lack of necessity beliefs; forgetfulness; concern beliefs | 4 (19) | 5 point Likert |
| MARS | 8 | English, Polish, French, Chinese | UK, France, Poland, China, Nigeria, Australia | Psychiatry | Self-administered | Yes | Medication adherence behaviour; attitude to psychotropic medication &towards taking medication; negative side effects | 3 (10) | Yes/no |
| MARS-10 | 1 | English, Spanish | US | Asthma | Self-administered | No | Adherence | 1 (10) | 5 point Likert Scale |
| MARS-5 | 8 | English, Arabic, Persian, Belgian, German, Norwegian | Jordan, Norway, Australia, Iran, Germany, Belgium | Asthma, severe mental disorder, TCS, stroke, COPD, HTN, CVD | Self-/ interviewer administered | No | Adherence | 1 (5) | 5 point Likert Scale |
| MARS-9 | 2 | English, Portuguese | US, Portugal | RA, chronic pain | Self-administered | No | Adherence | 1 (9) | 5 point Likert Scale |
| MASES | 2 | English, Persian | US, Iran | HTN | Self-administered | No | Confidence in taking blood pressure medications; confidence in ability to carry out the following tasks | 2 (26) | 3 point Likert Scale |
| MASES-R | 1 | English | US | HTN | Self-administered | No | Confidence in taking blood pressure medications; confidence in ability to carry out the following tasks | 2 (13) | 4 point Likert Scale |
| MASRI | 6 | English, Russian | US, Russia | BPO with OAB, SLE, UI | Self-administered | No | Frequency; correct timing of medication intake | 2 (12) |  |
| Medication adherence scale | 1 | Japanese | Japan | Chronic disease | Self-administered | No | Medication compliance; collaboration with healthcare providers; willingness to access and use information about medication; acceptance to take medication and how taking medication fits patient’s lifestyle | 4 (12) | 5 point Likert Scale |
| Medication adherence survey | 1 | English | Canada | Hemodialysis | Self-administered | No | Adherence; pill burden; timing of medications around dialysis; diet content; demographics; patient’s perception of how available their healthcare team was | 6 (23) | ? |
| MEDS | 1 | English | US | Diabetes, HTN, dyslipidemia | Self-administered | No | Side effects; addiction; cost; perceived need; unintentional nonadherence | 5 (16) | 5 point Likert Scale |
| MIS-A | 1 | French, English | France, UK | Asthma | Computer-assisted telephone interviews | Yes | Taking adherence; drug holidays; therapeutic coverage; overdosing; correct dosing | 5 (9) | Proportion, composite score |
| MMAS-4 | 18 | English, French, German, Indonesian, Chinese, Portuguese | Canada, Brazil, Germany, US, Indonesia, UK, Tanzania, Singapore | Glaucoma, T2DM, HTN, RA, TB, asthma, schizophrenia, CVD, alcohol use disorder, chronic disease | Self-administered | Yes | Adherence | 1 (4) | Yes/no |
| MMAS-7 | 1 | Arabic | UAE | HTN | Self-administered | No | Adherence | 1 (7) | Yes/No |
| MMAS-8 | 27 | Arabic, English, Spanish, Thai, Portuguese, Italian, French, Norwegian, Polish, Korean, Persian, German Chinese, Rukiga/ Ruyankore | Egypt, US, Germany, Libya, Spain, Brazil, Italy, Norway, South Korea, Poland, France, Iran, Uganda, Thailand, Saudi Arabia, Canada, Singapore | HTN, T2DM, gout, antiplatelet, psychiatric disorder, Parkinson’s disease, vitiligo, osteoporosis | Self-/ interviewer administered | Yes | Adherence | 1 (8) | Yes/no, 5 point Likert Scale |
| MMAS-9 | 1 | English | US | HIV/AIDS | Telephone administered | No | Adherence | 1 (9) | Yes/no, 5 point Likert Scale |
| MNPS | 1 | English | US | Chronic disease | Self-administered | No | Persistence |  |  |
| MOS General Adherence Scale | 1 | English | US | Chronic disease | Self-administered questionnaire | No | Adherence | 1 (5) | 6 point Likert Scale |
| MS-TAQ | 1 | English | US | MS | Self-administered | Yes | Barriers; side effects; coping strategies | 3 (30) | ? |
| OEOMA | 2 | English, Chinese | US, China | Osteoporosis | Self-administered | No | Outcome expectations | 1 (5) | 5 point Likert Scale |
| PEDIA scale | 1 | Portuguese | Brazil | HIV ART | Self-administered | No | Cognitive and routine problems; medication and health concerns; patient's fears and feelings | 3 (18) | 3 point Likert Scale |
| Pictographic self-efficacy scale | 1 | English | US | HIV/AIDS | Interviewer-administered | No | Adherence | ? | VAS |
| POP | 1 | Polish | Poland | Acute psychotic crisis | Self-administered | No | Compliance | 1 (1) | 5 point Likert Scale |
| PPQ | 1 | German, Dutch, English, French, Italian, Spanish, Swedish | France, Germany, Denmark, Italy, Spain, Sweden, UK, Netherlands | Psoriasis | Self-administered | No | Patient preference | 1 (10) | 4 point Likert Scale |
| ProMAS | 1 | Dutch | Netherlands | Chronic disease | Self-administered | Yes | Adherence | 1 (18) | Yes/no, True/false |
| PT/PP | 2 | English | US | Chronic disease | Self-administered | No | Pill-taking history for preceding week | 1 (2) | ? |
| QATOP | 1 | Portuguese | Portugal | Psoriasis | Self-administered | No | Description of the prescribed topical treatment; measurement of adherence and identification of treatment-associated variables | 2 (9) | Nominal, dichotomous, continuous, ordinal, 4 point Likert Scale |
| Question of Interest (Qis) | 1 | ? | Canada | Psoriasis | Self-administered | No | Psoriasis | Psoriasis | Psoriasis |
| SCI | 2 | English, Urdu | US, Pakistan | T1DM, T2DM | Self-administered | No | Adherence | 1 (14) | 5 point Likert Scale |
| SCI-R | 1 | Spanish, Catalan | Spain | Diabetes | Self-administered | No | Adherence | 1 (15) | 5 point Likert Scale |
| SCRAT | 1 | English | Scotland | Generic | Interviewer-administerd | No | Cognitive risk; physical risk | 2 (13) | 0, 1 or 2 objective scoring |
| SDSCA | 2 | English | US | T2DM | Self-administered | No | Medication-taking | 1 (1) | Numeric rating scale |
| SEAMS | 3 | Portuguese | Brazil | CAD, CHD | Self/ Interviewer-administered | No | Self-efficacy for taking medications in difficult circumstances; self-efficacy to continue to take the medication under uncertain circumstances | 2 (13) | 3 point Likert Scale |
| Self-efficacy scale | 1 | Persian | Iran | HTN | Self-administered | No | Self‑efficacy in different situations; self‑efficacy to overcome barriers | 2 (18) | 3 point Likert Scale |
| Self-report measures of adherence | 1 | English | US | Latent TB infection | Interviewer-administered | No | Self-reported medication adherence | 1 (?) | ? |
| Self-report on adherence | 1 | Portuguese | Brazil | HTN | Self-administered | No | Adherence | 1 (1) | Yes/no |
| SEOMA | 2 | English, Chinese | US, China | Osteoporosis | Self-administered | No | Self-efficacy | 1 (14) | 10 point Likert Scale |
| SERAD questionnaire | 1 | Spanish | Spain | ART | Interviewer-administered questionnaire | No | ? | ? | ? |
| SICT | 1 | English | US, UK | Iron chelation therapy | Self-administered | No | Satisfaction; adherence | 2 (28) | 5 point Likert Scale |
| *Sidorkiewicz et al*^a^ | 1 | French | France | Chronic disease | ? | No | ? | ? | ? |
| SMAQ | 2 | Spanish, English | Spain, US | ART, renal transplant | Self-administered | No | Adherence | 1 (6) | Yes/no, categorical |
| SOC Questionnaire | 2 | English | US, Canada, France, Germany, Italy | Chronic disease, HIV ART | Self-administered | No | Stage of change | 1 (2) | Categorical |
| SPNS adherence survey | 1 | English | US | HIV ART | Self-administered | No | Adherence | 1 (3) | 4 point Likert Scale, recall |
| SRSI | 4 | English, Spanish | US | HIV, T2DM | Self-administered | Yes | Adherence | 1 (3) | Categorical, 5/6 point Likert |
| TAI | 1 | Spanish | Spain | Asthma, COPD | Self-administered | No | Patient domain; HCP domain | 2 (12) | 5 point Likert, dichotomous |
| *Tan et al*^a^ |  | Chinese | China | CKD | Self-administered | No | Basic knowledge on medication; knowledge about Chinese medicine decoction; knowledge about taking Chinese medicine; medication beliefs; medication behavior | 5 (26) | ? |
| TASHP | 1 | English | US | OCD | Rater-administered | No | CBT; medications | 2 (14) | Yes/no, categorical, numerical |
| TAS-P | 1 | Chinese | China | HTN | Self-administered | No | Adherence | 1 (25) | 5 point Likert Scale |
| TTAQ | 1 | German, Dutch, English, French, Italian, Spanish, Swedish | Denmark, France, Germany, Italy, Spain, Sweden, Netherlands, UK | Psoriasis | Self-administered | No | Benefit to patients; information, communication and relationship towards the doctor; satisfaction with the treatment | 3 (59) | 4 point Likert Scale |
| *Turcu-știolică et al*^a^ | 1 | Romanian | Romania | COPD | Self-administered | No | Adherence; factors; quality of life | ? | ? |
| VAS | 4 | English, Spanish, Thai | US, Thailand | HIV | Self-administered | Yes | Adherence (4 weeks) | 1 (1) | VAS |
| VERITAS-PRN | 1 | French, English | Canada, US | Haemophilia | Self-administered | No | Treat; time; dose; plan; remember; communicate | 6 (24) | 5 point Likert Scale |
| VERITAS-Pro | 1 | French, English | Canada, US | Haemophilia | Self-administered | No | Time, dose, plan, remember, skip, communicate | 6 (24) | ? |
| *Voils et al*^a^ | 4 | English, Spanish | US, Singapore | HTN, T2DM, acute coronary syndrome, hyperlipidemia | Self-administered | No | Extent of nonadherence; reasons for nonadherence | 2 (28) | 5 point Likert Scale |
| *Vreeman et al*^a^ | 2 | English | Kenya, South Africa, Thailand | HIV | Self-administered | No | Adherence | 1 (10) | ? |
| Web-Ad-Q Questionnaire | 1 | ? | Brazil | ART | Electronic questionnaire | Yes | Adherence | 1 (3) | Yes/no |
| *Wilson et al*^a^ | 3 | English,  isiXhosa | US, South Africa | ART | Self-administered | No | Adherence | 1 (3) | 6 point Likert, numerical |

?, Not reported

^a^ PROMs without proper names are labelled based on the last name of the first author who developed the instrument

**Abbreviations:**

AAAQ: Adult Asthma Adherence Questionnaire; AACTG: Adult AIDS Clinical Trials Group; AAS: Antidepressant Adherence Scale; ABQ: Adherence Barrier Questionnaire; ADEOS: Adherence Evaluation of Osteoporosis Treatment Questionnaire; AMBAS: Antipsychotic Medication Beliefs and Attitudes Scale; APRQ: Adherence to Pulmonary Rehabilitation Questionnaire; ARMS: Adherence to Refills and Medications Scale; ASK: Adherence Starts with Knowledge questionnaire; ASRQ: Adherence self-report questionnaire; BAASIS: Basel Assessment of Adherence to Immunosuppressive Medications Scale; BEMIB: Brief Evaluation of Medication Influences and Beliefs; BERMA: Beliefs Related to Medication Adherence; BMAS: Brief Medication Adherence Scale; BMCS: Beliefs about Medication Compliance Scale; BMQ: Brief Medication Questionnaire; CASE: Center for Adherence Support Evaluation; CDCI: Chronic Disease Compliance Instrument; CEAT-VIH: *Cuestionario para la Evaluacio´ n de la Adhesio´ n al Tratamiento Antirretroviral en Personas con Infeccio´ n por VIH y Sida’’*;CoSMO: Cohort Study of Medication Adherence Among Older Adults; CQR: Compliance Questionnaire on Rheumatology; DAI: Drug Attitude Inventory; DAMS: Diagnostic Adherence to Medication Scale; DMQ: Diabetes Management Questionnaire; DMSS: Diabetes Medication Self-efficacy Scale; DRAW: Drug Adherence Work-Up Tool; EBAS: Environmental Barriers to Adherence Scale; EDSQ: Eye-Drop Satisfaction Questionnaire; ESRD-AQ: End-Stage Renal Disease Adherence Questionnaire; GMAS: General Medicine Adherence Scale; GTCAT: Glaucoma Treatment Compliance Assessment Tool; HIV-IM: HIV Intention Measure; HIV-SQUAD: HIV Symptom Quality of Life Adherence Questionnaire; IADMAS: Iraqi Anti-Diabetic Medication Adherence Scale; IRT: Item Response Theory; ITAS: Immunosuppressant Therapy Adherence Scale; ITBS: Immunosuppressant Therapy Barrier Scale; Lasso- Least absolute shrinkage and selection operator; MAQ: Medication Adherence Questionnaire; MAR-Scale: Medication Adherence Reasons Scale MARS: Medication Adherence Rating Scale; MARS-5/9/10: 5/9/10-item Medication Adherence Report Scale; MASES: Medication Adherence Self-efficacy Scale; MASRI: Medication Adherence Self-Report Inventory; MEDS: Medication Adherence Estimation and Differentiation Scale; MIS-A: Medication Intake Survey-Asthma; MMAS: Morisky Medication Adherence Scale; MNPS: Medication Nonpersistence Scale; MOS: Medical Outcomes Study; MS-TAQ: Multiple Sclerosis Treatment Adherence Questionnaire; OEOMA: Outcome Expectations for Osteoporosis Medication Adherence Scale; PEDIA: Perceived Barriers to Antiretroviral Therapy Adherence Scale; POP: Patient Rating of Compliance Scale; PPQ: Patient Preference Questionnaire; ProMAS: Probabilistic Medication Adherence Scale: PT/PP: No. of pills taken/prescribed; QATOP: Questionnaire for Adherence with Topical Treatments in Psoriasis; SCI: Self-Care Inventory; SCRAT: Strathclyde Compliance Risk Assessment Tool; SDSCA: Summary of Diabetes Self-care Activities; SEAMS: Self-Efficacy for Appropriate Medication Adherence Scale; SEOMA: Self-Efficacy for Osteoporosis Medication Adherence Scale; SERAD: Self-Reported Adherence questionnaire; SICT: Satisfaction with iron chelation therapy; SMAQ: Simplified Medication Adherence Questionnaire; SOC: Stages of change model; SPNS: Special Projects of National Significance adherence tool; SRSI: Self-rating scale item; TAI: Test of the Adherence to Inhalers; TAS-P: Treatment Adherence Survey-Patient Version; TASHP: Therapeutic Adherence Scale for Hypertensive Patients; TTAQ: Topical Therapy Adherence Questionnaire; VAS: Visual analogue scale; VERITAS-PRN: Validated Hemophilia Regimen Treatment Adherence Scale – On-Demand; VERITAS-Pro: Validated Hemophilia Regimen Treatment Regimen Treatment Adherence Scale-Prophylaxis
