# Supplementary material for: Measurement Properties of Existing Patient-Reported Outcome Measures on Medication Adherence: Systematic Review
Source: J Med Internet Res. 2020 Oct 9;22(10):e19179. doi: 10.2196/19179 (PMC7584986; doi:10.2196/19179)
Supplement: Multimedia Appendix 3 [file jmir_v22i10e19179_app3.docx]

**Supplementary Table 3**. Assessment of psychometric properties of PROMs.

|  |  | **Methodological quality of studies/ quality of measurement properties of PROM** | | | | | | | | | |
| --- | --- | --- | --- | --- | --- | --- | --- | --- | --- | --- | --- |
| **PROM** | **Reference** | **PROM development** | **Content validity** | **Structural validity** | **Internal consistency** | **Cross-cultural validity\ Measurement invariance** | **Reliability** | **Measurement error** | **Criterion validity** | **Hypotheses testing for construct validity** | **Responsiveness** |
| AAAQ | Schatz et al., 2013 ^53^ | 0 | ?/adequate | ?/adequate | -/doubtful | 0 | 0 | 0 | 0 | -/adequate | +/adequate |
| AACTG | Buscher et al., 2011^54^ | 0 | 0 | 0 | 0 | 0 | 0 | 0 | 0 | +/adequate | 0 |
| AACTG ^b^ | Gonzalez et al., 2013 ^55^ | 0 | 0 | 0 | 0 | 0 | 0 | 0 | 0 | -/doubtful | 0 |
| AACTG ^b^ | Kalichman et al., 2007 ^56^ | 0 | 0 | 0 | 0 | 0 | 0 | 0 | 0 | +/adequate | 0 |
| AACTG ^b^ | Reynolds et al., 2007 ^57^ | 0 | 0 | ?/adequate | +/very good | 0 | 0 | 0 | 0 | +/adequate | 0 |
| AACTG ^b^ | Simoni et al., 2014^58^ | 0 | 0 | 0 | 0 | 0 | 0 | 0 | 0 | ?/doubtful | 0 |
| AACTG ^b^ | Smith et al., 2007 ^59^ | 0 | 0 | 0 | 0 | 0 | 0 | 0 | 0 | ?/doubtful | 0 |
| AACTG ^b^ | van der Boogaard et al., 2011 ^60^ | 0 | 0 | 0 | 0 | 0 | 0 | 0 | 0 | 0 | +/doubtful |
| AACTG ^b^ | Chesney et al., 2000 ^61^ | 0 | ?/inadequate | ?/inadequate | 0 | 0 | 0 | 0 | 0 | +/doubtful | 0 |
| AAS | Gabriel et al., 2010 ^62^ | inadequate | ?/adequate | 0 | -/inadequate | 0 | 0 | 0 | 0 | -/adequate | 0 |
| ABQ | Müller et al., 2015 ^63^ | inadequate | 0 | ?/adequate | +/very good | 0 | 0 | 0 | 0 | -/adequate | 0 |
| ABQ-HIV^b^ | Mueller et al., 2018 ^64^ | 0 | 0 | 0 | +/very good | 0 | 0 | 0 | 0 | +/very good | +/very good |
| Adherence tool for CML | Daouphars et al., 2013 ^65^ | inadequate | 0 | 0 | -/very good | 0 | 0 | 0 | 0 | -/inadequate | -/doubtful |
| ADEOS-12 | Breuil et al., 2012 ^66^ | 0 | 0 | 0 | 0 | 0 | 0 | 0 | 0 | +/very good | +/very good |
| AMBAS | Martins et al., 2019 ^67^ | inadequate | +/doubtful | -/adequate | +/very good | 0 | 0 | 0 | 0 | +/adequate | 0 |
| APRQ | Ayiesah et al., 2009 ^68^ | doubtful | +/doubtful | ?/adequate | +/inadequate | 0 | 0 | 0 | 0 | 0 | 0 |
| ARMS | Kripalani et al., 2017 ^69^ | doubtful | +/doubtful | ?/adequate | +/very good | 0 | ?/doubtful | 0 | 0 | +/adequate | 0 |
| ARMS-7 | Gokdogan et al., 2009 ^70^ | 0 | ?/doubtful | -/very good | +/very good | 0 | +/adequate | 0 | 0 | 0 | 0 |
| ASK-12 | Matza et al., 2017 ^71^ | 0 | 0 | ?/adequate | +/very good | 0 | +/adequate | 0 | 0 | +/very good | 0 |
| ASK-20 | Atsuta et al., 2008 ^72^ | 0 | 0 | 0 | +/inadequate | 0 | 0 | 0 | 0 | +/adequate | 0 |
| ASK-20 | Hahn et al., 2008 ^73^ | adequate | +/adequate | ?/adequate | +/inadequate | 0 | 0 | 0 | 0 | +/adequate | -/adequate |
| ASK-20 | Matza et al., 2008 ^74^ | 0 | 0 | 0 | +/inadequate | 0 | +/adequate | 0 | 0 | -/very good | 0 |
| ASRQ | Zeller et al, 2008^75^ | 0 | +/doubtful | 0 | 0 | 0 | -/inadequate | 0 | 0 | 0 | -/adequate |
| Attitudes to mesalamine questionnaire | Moss et al., 2014 ^76^ | inadequate | 0 | 0 | +/inadequate | 0 | 0 | 0 | 0 | +/very good | +/very good |
| *Axelsson et al*^c^ | Axelsson et al., 2016 ^77^ | inadequate | 0 | ?/adequate | +/very good | 0 | 0 | 0 | 0 | +/doubtful | 0 |
| BAASIS | Marsicano Ede et al., 2013 ^78^ | 0 | +/adequate | ?/adequate | +/inadequate | ^a^ | +/very good | 0 | 0 | +/adequate | 0 |
| BEMIB | Dolder et al., 2004 ^79^ | inadequate | ?/doubtful | ?/adequate | -/inadequate | 0 | ?/doubtful | 0 | 0 | -/inadequate | +/adequate |
| BERMA | McDonald-Miszczak et al., 2014 ^80^ | doubtful | ?/inadequate | ?/inadequate | +/doubtful | 0 | 0 | 0 | 0 | +/doubtful | 0 |
| BMAS | Chui et al., 2018 ^81^ | inadequate | 0 | ?/adequate | -/inadequate | 0 | +/adequate | 0 | 0 | +/adequate | 0 |
| BMCS | Bennett et al., 1997 ^82^ | inadequate | +/doubtful | ?/adequate | +/very good | 0 | 0 | 0 | 0 | 0 | 0 |
| BMQ^b^ | Sriwarakorn et al., 2010 ^83^ | 0 | ?/doubtful | 0 | 0 | 0 | +/adequate | 0 | 0 | 0 | +/doubtful |
| BMQ^b^ | Svarstad et al.,1999 ^84^ | inadequate | 0 | 0 | 0 | 0 | 0 | 0 | 0 | +/adequate | +/adequate |
| BMQ^b^ | van den Boogaard et al., 2011 ^60^ | 0 | 0 | 0 | 0 | 0 | 0 | 0 | 0 | 0 | -/doubtful |
| BMQ^b^ | Choo et al., 1999 ^85^ | 0 | 0 | 0 | 0 | 0 | 0 | 0 | 0 | +/adequate | 0 |
| CASE adherence index | Kerr et al., 2012 ^86^ | 0 | 0 | 0 | 0 | ^a^ | ?/inadequate | 0 | 0 | +/adequate | -/adequate |
| CASE adherence index | Mannheimer et al., 2006 ^87^ | 0 | 0 | 0 | 0 | 0 | 0 | 0 | 0 | +/inadequate | +/inadequate |
| CDCI | Kyngäs et al., 2000 ^88^ | very good | +/very good | ?/adequate | +/very good | ^a^ | 0 | 0 | 0 | +/inadequate | 0 |
| CDCI-asthma^b^ | Wales et al., 2011 ^89^ | 0 | 0 | ?/adequate | -/very good | 0 | 0 | 0 | 0 | -/very good | 0 |
| CDCI-diabetes ^b^ | Fernandes et al., 2011 ^90^ | 0 | 0 | ?/adequate | +/very good | 0 | 0 | 0 | 0 | -/very good | 0 |
| CEAT-VIH | Dima et al., 2013 ^91^ | 0 | +/adequate | 0 | +/inadequate | 0 | 0 | 0 | 0 | +/doubtful | 0 |
| *Chaiyachati et al*^c^ | Chaiyachati et al., 2011 ^92^ | 0 | 0 | 0 | 0 | 0 | 0 | 0 | 0 | 0 | -/doubtful |
| Compliance assessment | Kampman et al., 2001 ^93^ | 0 | 0 | 0 | ?/inadequate | 0 | 0 | 0 | 0 | +/adequate | 0 |
| CoSMO self-report tool | Krousel-Wood et al., 2013 ^94^ | inadequate | 0 | ?/inadequate | 0 | 0 | 0 | 0 | 0 | +/adequate | +/adequate |
| CQR | de Klerk et al., 1999^95^ | adequate | +/adequate | 0 | +/inadequate | 0 | +/adequate | 0 | 0 | -/adequate | +/very good |
| CQR | Salt et al., 2012 ^96^ | 0 | 0 | ?/adequate | +/very good | 0 | -/adequate | 0 | 0 | +/very good | 0 |
| CQR (KCQR) | Lee et al., 2011 ^97^ | 0 | +/adequate | 0 | 0 | ^a^ | +/doubtful | 0 | 0 | -/doubtful | 0 |
| CQR-T (CQR) | Cinar et al., 2015 ^98^ | 0 | +/adequate | ?/adequate | +/very good | 0 | -/adequate | 0 | 0 | +/adequate | 0 |
| CQR5 | Hughes et al., 2013 ^99^ | 0 | 0 | +/very good | +/doubtful | 0 | 0 | 0 | 0 | +/adequate | -/adequate |
| *Da et al*^c^ | Da et al., 2018 ^100^ | 0 | 0 | 0 | ?/inadequate | 0 | 0 | 0 | 0 | -/doubtful | -/adequate |
| DAI | Hogan et al., 1983 ^101^ | inadequate | 0 | 0 | +/inadequate | 0 | ?/inadequate | 0 | 0 | +/adequate | 0 |
| DAI-10 | Friemann et al., 2013 ^102^ | 0 | 0 | 0 | +/doubtful | 0 | 0 | 0 | 0 | +/adequate | -/adequate |
| DAI-10 | Kikkert et al., 2011 ^103^ | 0 | 0 | 0 | 0 | 0 | 0 | 0 | 0 | -/very good | -/adequate |
| DAI-9 | Stjernsward et al., 2013 ^104^ | 0 | 0 | ?/adequate | 0 | 0 | -/inadequate | 0 | 0 | -/doubtful | 0 |
| DAMS | Garfield et al., 2012 ^105^ | adequate | +/adequate | 0 | 0 | 0 | 0 | 0 | 0 | +/very good | 0 |
| *Demirtas et al*^c^ | Demirtas et al., 2017 ^106^ | adequate | +/adequate | ?/very good | +/very good | 0 | ?/doubtful | 0 | 0 | +/doubtful | 0 |
| DMARS-4 | Jacobsen et al., 2009 ^107^ | 0 | ?/doubtful | 0 | +/very good | ^a^ | 0 | 0 | 0 | -/doubtful | 0 |
| DMQ | Mehta et al., 2015 ^108^ | inadequate | ?/doubtful | 0 | +/very good | 0 | -/doubtful | 0 | 0 | +/adequate | 0 |
| DMSS | Sleath et al., 2016 ^109^ | inadequate | 0 | 0 | +/inadequate | 0 | 0 | 0 | 0 | ?/doubtful | 0 |
| DMSS (CDMSS) | Zheng et al., 2018 ^110^ | 0 | +/adequate | ?/adequate | +/inadequate | ^a^ | +/adequate | 0 | 0 | +/adequate | -/adequate |
| EBAS | Irvine et al., 1990 ^111^ | 0 | 0 | 0 | +/very good | 0 | 0 | 0 | 0 | +/inadequate | 0 |
| EDSQ | Regnault et al., 2010 ^112^ | 0 | 0 | ?/adequate | +/very good | 0 | 0 | 0 | 0 | ?/doubtful | 0 |
| EDSQ | Nordmann et al., 2007 ^113^ | doubtful | +/doubtful | 0 | 0 | 0 | 0 | 0 | 0 | 0 | 0 |
| ESRD-AQ | Kim et al., 2010 ^114^ | inadequate | +/adequate | 0 | 0 | 0 | +/inadequate | 0 | 0 | 0 | 0 |
| ESRD-AQ (PESRD-EQ) | Poveda et al., 2016 ^115^ | 0 | +/adequate | 0 | 0 | ^a^ | +/doubtful | 0 | 0 | -/adequate | 0 |
| Every Visit Adherence Questionnaire | Fairley et al., 2005 ^116^ | 0 | 0 | 0 | 0 | 0 | 0 | 0 | 0 | +/inadequate | 0 |
| Five-dimension adherence model | Kandrotaite et al., 2013 ^117^ | inadequate | 0 | 0 | 0 | 0 | 0 | 0 | 0 | 0 | +/very good |
| *Fredericksen et al*^c^ | Fredericksen et al., 2019 ^118^ | doubtful | +/very good | 0 | 0 | 0 | +/very good | 0 | 0 | 0 | 0 |
| General adherence tendency measure | Zongo et al., 2016 ^119^ | inadequate | 0 | 0 | 0 | 0 | 0 | 0 | 0 | -/adequate | -/adequate |
| GMAS | Naqvi et al., 2019 ^120^ | 0 | 0 | +/very good | +/very good | 0 | -/adequate | 0 | 0 | +/adequate | +/adequate |
| GMAS | Naqvi et al., 2019 ^121^ | 0 | ?/inadequate | +/very good | +/very good | ^a^ | +/adequate | 0 | 0 | +/adequate | ?/doubtful |
| GMAS | Naqvi et al., 2018 ^122^ | doubtful | +/very good | +/very good | +/very good | 0 | -/adequate | 0 | 0 | +/doubtful | +/doubtful |
| *Godin et al*^c^ | Godin et al., 2003 ^123^ | doubtful | ?/doubtful | 0 | 0 | 0 | 0 | 0 | 0 | -/doubtful | +/doubtful |
| *Godin et al* ^b c^ | Zongo et al., 2016 ^119^ | 0 | 0 | 0 | 0 | 0 | 0 | 0 | 0 | -/adequate | -/adequate |
| GTCAT | Mansberger et al., 2013 ^124^ | inadequate | ?/adequate | ?/doubtful | -/very good | 0 | ?/inadequate | 0 | 0 | +/very good | 0 |
| GTCAT (reduced) | Abe et al., 2018 ^125^ | 0 | ?/adequate | -/doubtful | -/very good | ^a^ | +/adequate | 0 | 0 | 0 | 0 |
| GTCAT (reduced) | Barker et al., 2019 ^126^ | 0 | 0 | ?/adequate | -/very good | 0 | 0 | 0 | 0 | -/adequate | 0 |
| Hill Bone Scale (HBMA-K) | Song et al., 2011 ^127^ | 0 | ?/doubtful | ?/adequate | +/very good | ^a^ | 0 | 0 | 0 | +/very good | 0 |
| Hill-Bone Scale | Kim et al., 2000 ^18^ | adequate | +/adequate | ?/adequate | +/very good | 0 | 0 | 0 | 0 | -/very good | 0 |
| Hill-Bone Scale | Koschack et al., 2010 ^128^ | 0 | 0 | ?/adequate | +/adequate | ^a^ | 0 | 0 | 0 | -/doubtful | 0 |
| Hill-Bone Scale | Krousel-Wood et al., 2005 ^129^ | 0 | 0 | ?/adequate | -/very good | 0 | 0 | 0 | 0 | 0 | 0 |
| Hill-Bone Scale | Nogueira-Silva et al., 2016 ^130^ | 0 | +/very good | 0 | 0 | ^a^ | 0 | 0 | 0 | 0 | 0 |
| Hill-Bone Scale (modified) | Alsolami et al., 2013 ^131^ | 0 | ?/doubtful | 0 | -/inadequate | ^a^ | 0 | 0 | 0 | 0 | 0 |
| HIV-IM | Nelsen et al., 2012 ^132^ | doubtful | +/adequate | ?/adequate | +/very good | 0 | 0 | 0 | 0 | +/doubtful | 0 |
| HIV-SQUAD | Spire et al., 2009^133^ | inadequate | 0 | ?/adequate | +/doubtful | 0 | 0 | 0 | 0 | +/adequate | 0 |
| IADMAS | Mikhael et al., 2019 ^134^ | doubtful | ?/doubtful | 0 | +/very good | ^a^ | ?/adequate | 0 | 0 | +/very good | +/adequate |
| IRT-30 | Wohl et al., 2018 ^135^ | adequate | +/adequate | ?/adequate | 0 | 0 | 0 | 0 | 0 | ?/doubtful | +/adequate |
| ITAS | Jesus-Nunes et al., 2018 ^136^ | 0 | 0 | ?/adequate | +/inadequate | ^a^ | 0 | 0 | 0 | +/adequate | 0 |
| ITAS | Wilks et al., 2010 ^137^ | 0 | 0 | ?/adequate | +/inadequate | 0 | 0 | 0 | 0 | -/adequate | 0 |
| ITBS | Chisholm et al., 2005 ^138^ | doubtful | +/adequate | ?/adequate | +/very good | 0 | 0 | 0 | 0 | +/very good | +/very good |
| *Kennedy et al*^c^ | Kennedy et al., 2000 ^139^ | doubtful | +/adequate | 0 | 0 | 0 | 0 | 0 | 0 | 0 | 0 |
| *Kerr et al*^c^ | Kerr et al., 2008 ^140^ | inadequate | 0 | 0 | 0 | 0 | 0 | 0 | 0 | -/inadequate | -/adequate |
| Lasso-10 | Wohl et al., 2018 ^135^ | adequate | +/adequate | ?/adequate | 0 | 0 | 0 | 0 | 0 | ?/doubtful | +/adequate |
| Long-Term Medication Behaviour Self-Efficacy Scale | De Geest et al., 1994 ^141^ | inadequate | ?/doubtful | 0 | 0 | 0 | 0 | 0 | 0 | 0 | 0 |
| M-DRAW | Lee et al., 2017 ^142^ | 0 | 0 | 0 | +/inadequate | 0 | 0 | 0 | 0 | -/adequate | +/adequate |
| M-DRAW | Lee et al., 2017 ^143^ | 0 | 0 | ?/doubtful | +/inadequate | 0 | 0 | 0 | 0 | -/adequate | +/adequate |
| MAQ | Sadakathulla et al., 2019 ^144^ | inadequate | ?/doubtful | 0 | +/inadequate | ^a^ | +/adequate | 0 | 0 | 0 | 0 |
| MAR-Scale | Unni et al., 2015 ^145^ | doubtful | ?/inadequate | ?/adequate | -/very good | 0 | -/inadequate | 0 | 0 | +/inadequate | 0 |
| MAR-Scale (revised) | Unni et al., 2014 ^146^ | adequate | +/adequate | ?/adequate | +/very good | 0 | 0 | 0 | 0 | +/adequate | 0 |
| MAR-Scale (revised) | Unni et al., 2019 ^147^ | 0 | 0 | 0 | +/very good | 0 | 0 | 0 | 0 | 0 | 0 |
| MARS | Fialko et al., 2008 ^148^ | 0 | 0 | ?/adequate | -/doubtful | 0 | 0 | 0 | 0 | -/inadequate | 0 |
| MARS | Fond et al., 2017 ^149^ | 0 | 0 | -/very good | -/very good | 0 | 0 | 0 | 0 | -/inadequate | 0 |
| MARS | Friemann et al., 2013 ^102^ | 0 | 0 | 0 | -/doubtful | 0 | 0 | 0 | 0 | +/adequate | +/adequate |
| MARS | Kao et al., 2010 ^150^ | 0 | +/doubtful | ?/adequate | +/very good | ^a^ | ?/inadequate | 0 | 0 | +/very good | 0 |
| MARS | Owie et al., 2018 ^151^ | 0 | 0 | ?/doubtful | +/very good | 0 | 0 | 0 | 0 | -/very good | 0 |
| MARS | Sowunmi et al., 2019 ^152^ | 0 | +/doubtful | ?/adequate | +/doubtful | 0 | 0 | 0 | 0 | -/very good | 0 |
| MARS | Thompson et al., 2000 ^153^ | inadequate | 0 | ?/adequate | +/inadequate | 0 | ?/inadequate | 0 | 0 | +/very good | 0 |
| MARS | Zemmour et al., 2016 ^154^ | 0 | ?/inadequate | +/very good | -/very good | ^a^ | 0 | 0 | 0 | -/adequate | 0 |
| MARS-10 (MARS-A) | Cohen et al., 2009 ^155^ | 0 | ?/inadequate | ?/adequate | +/very good | ^a^ | 0 | 0 | 0 | +/adequate | +/adequate |
| MARS-5 | Alsous et al., 2017 ^156^ | 0 | +/adequate | ?/adequate | +/very good | ^a^ | +/adequate | 0 | 0 | ?/inadequate | 0 |
| MARS-5 | Garcia-Marcos et al., 2016 ^157^ | 0 | 0 | 0 | 0 | 0 | 0 | 0 | 0 | +/adequate | +/adequate |
| MARS-5 | Jonsdottir et al., 2010 ^158^ | 0 | 0 | 0 | 0 | 0 | 0 | 0 | 0 | +/adequate | 0 |
| MARS-5 | Lee et al., 2019 ^159^ | 0 | 0 | +/very good | +/very good | 0 | 0 | 0 | 0 | -/inadequate | 0 |
| MARS-5 | Lin et al., 2018 ^160^ | 0 | 0 | ?/very good | +/inadequate | 0 | 0 | 0 | 0 | +/very good | 0 |
| MARS-5 | Tommelein et al., 2014 ^161^ | 0 | 0 | 0 | +/very good | 0 | 0 | 0 | 0 | -/inadequate | -/inadequate |
| MARS-5 | van de Steeg et al., 2009 ^162^ | 0 | 0 | 0 | 0 | 0 | 0 | 0 | 0 | 0 | -/adequate |
| MARS-5 (MARS-D) | Mahler et al., 2010 ^163^ | 0 | +/adequate | 0 | -/inadequate | ^a^ | -/adequate | 0 | 0 | -/very good | 0 |
| MARS-9 (MARS-9RA) | Salt et al., 2012 ^96^ | 0 | 0 | ?/adequate | +/very good | 0 | -/adequate | 0 | 0 | +/very good | 0 |
| MARS-9 (MARS-P9) | Sampaio et al., 2019^164^ | 0 | +/adequate | ?/adequate | +/very good | ^a^ | 0 | 0 | 0 | -/adequate | 0 |
| MASES | Ogedegbe et al., 2013 ^165^ | inadequate | +/doubtful | 0 | +/very good | 0 | ?/inadequate | 0 | 0 | -/adequate | 0 |
| MASES | Saffari et al., 2015 ^166^ | 0 | +/very good | ?/adequate | +/very good | ^a^ | -/doubtful | 0 | 0 | +/very good | 0 |
| MASES-R | Fernandez et al., 2008 ^166^ | 0 | 0 | ?/adequate | +/inadequate | 0 | -/doubtful | 0 | 0 | -/adequate | 0 |
| MASRI | Andy et al., 2015 ^167^ | 0 | 0 | 0 | 0 | 0 | 0 | 0 | 0 | +/very good | +/adequate |
| MASRI | Koneru et al., 2007 ^168^ | 0 | 0 | 0 | +/very good | 0 | +/doubtful | 0 | 0 | +/very good | 0 |
| MASRI | Kosilov et al., 2018 ^169^ | 0 | 0 | 0 | 0 | 0 | 0 | 0 | 0 | +/very good | +/very good |
| MASRI | Kosilov et al., 2017 ^170^ | 0 | 0 | 0 | 0 | 0 | 0 | 0 | 0 | +/adequate | +/adequate |
| MASRI | Kosilov et al., 2017 ^171^ | 0 | 0 | 0 | 0 | 0 | 0 | 0 | 0 | +/very good | +/very good |
| MASRI | Kosilov et al., 2017 ^172^ | 0 | 0 | 0 | 0 | 0 | 0 | 0 | 0 | +/very good | +/very good |
| Medication adherence scale | Ueno et al., 2018 ^173^ | inadequate | +/doubtful | -/very good | +/very good | 0 | 0 | 0 | 0 | -/adequate | 0 |
| Medication adherence survey | Battistella et al., 2016 ^174^ | adequate | +/adequate | 0 | 0 | 0 | 0 | 0 | 0 | +/inadequate | 0 |
| MEDS | Athavale et al. 2019 ^175^ | doubtful | ?/doubtful | +/very good | +/very good | 0 | 0 | 0 | 0 | +/inadequate | -/very good |
| MIS-A | Dima et al., 2017 ^176^ | adequate | +/adequate | 0 | 0 | 0 | -/adequate | 0 | 0 | +/adequate | 0 |
| MMAS-4 | Cate et al., 2015 ^177^ | 0 | 0 | 0 | 0 | 0 | -/inadequate | 0 | 0 | -/doubtful | 0 |
| MMAS-4 | Cohen et al., 2010 ^178^ | 0 | 0 | 0 | 0 | 0 | 0 | 0 | 0 | -/inadequate | 0 |
| MMAS-4 | Guénette et al., 2005 ^179^ | 0 | 0 | 0 | 0 | 0 | -/inadequate | 0 | 0 | -/doubtful | 0 |
| MMAS-4 | Morisky et al., 1986 ^180^ | 0 | 0 | ?/adequate | -/very good | 0 | 0 | 0 | 0 | +/very good | -/very good |
| MMAS-4 | Salt et al., 2012 ^96^ | 0 | 0 | ?/adequate | -/very good | 0 | +/adequate | 0 | 0 | +/very good | 0 |
| MMAS-4 | Shalansky et al., 2004 ^181^ | 0 | 0 | 0 | -/adequate | 0 | 0 | 0 | 0 | +/adequate | -/doubtful |
| MMAS-4 | van de Steeg et al., 2009 ^162^ | 0 | 0 | 0 | 0 | 0 | 0 | 0 | 0 | 0 | -/adequate |
| MMAS-4 | van den Boogaard, et al., 2011 ^60^ | 0 | 0 | 0 | 0 | 0 | 0 | 0 | 0 | 0 | -/doubtful |
| MMAS-4 (asthma medication) ^b^ | Brooks et al., 1994 ^182^ | 0 | 0 | 0 | -/very good | 0 | ?/inadequate | 0 | 0 | 0 | +/very good |
| MMAS-4 (inhaler) ^b^ | Brooks et al., 1994 ^182^ | 0 | 0 | 0 | +/very good | 0 | ?/inadequate | 0 | 0 | 0 | +/very good |
| MMAS-4 (inhaler) ^b^ | Erickson et al., 2001 ^183^ | 0 | 0 | 0 | +/inadequate | 0 | 0 | 0 | 0 | -/adequate | 0 |
| MMAS-4 (MAQ) | Kikkert et al., 2001 ^103^ | 0 | 0 | 0 | 0 | 0 | 0 | 0 | 0 | +/very good | +/adequate |
| MMAS-4 (MGLS) | Kristina et al., 2019 ^184^ | 0 | +/doubtful | 0 | -/very good | ^a^ | ?/adequate | 0 | 0 | +/very good | -/very good |
| MMAS-4 (MGLS) | Wang et al., 2012 ^185^ | 0 | inadequate | ?/adequate | -/very good | ^a^ | 0 | 0 | 0 | 0 | 0 |
| MMAS-4 (Morisky Green Scale) | Koschack et al., 2010 ^128^ | 0 | 0 | ?/adequate | -/adequate | ^a^ | 0 | 0 | 0 | -/doubtful | 0 |
| MMAS-4 (Morisky-Green Test) | Prado et al., 2007 ^186^ | 0 | 0 | 0 | 0 | 0 | 0 | 0 | 0 | +/doubtful | -/doubtful |
| MMAS-4 (SR-4) | Zongo et al.,2016 ^119^ | 0 | 0 | 0 | 0 | 0 | 0 | 0 | 0 | -/adequate | -/adequate |
| MMAS-4 (SRMTS) | Jerant et al., 2008 ^187^ | 0 | 0 | 0 | -/inadequate | 0 | 0 | 0 | 0 | -/doubtful | 0 |
| MMAS-7 | Fahey et al., 2006 ^188^ | 0 | 0 | 0 | 0 | ^a^ | 0 | 0 | 0 | +/adequate | 0 |
| MMAS-8 | Ali et al.,2016 ^189^ | 0 | 0 | 0 | +/inadequate | 0 | 0 | 0 | 0 | -/very good | 0 |
| MMAS-8 | Ambarish et al., 2013 ^190^ | 0 | 0 | 0 | -/doubtful | 0 | 0 | 0 | 0 | 0 | 0 |
| MMAS-8 | Ashur et al., 2015 ^191^ | 0 | 0 | 0 | +/doubtful | 0 | ?/inadequate | 0 | 0 | +/very good | 0 |
| MMAS-8 | De Las Cuevas et al., 2015 ^192^ | 0 | 0 | -/very good | +/doubtful | 0 | 0 | 0 | 0 | +/doubtful | 0 |
| MMAS-8 | de Oliveira et al., 2014 ^193^ | 0 | +/adequate | 0 | -/very good | 0 | ?/adequate | 0 | 0 | +/adequate | +/adequate |
| MMAS-8 | Dibonaventura et al., 2014 ^194^ | 0 | 0 | ?/very good | -/very good | 0 | 0 | 0 | 0 | +/adequate | 0 |
| MMAS-8 | Fabbrini et al., 2013 ^195^ | 0 | +/very good | 0 | 0 | 0 | 0 | 0 | 0 | +/doubtful | 0 |
| MMAS-8 | George et al., 2006 ^196^ | 0 | 0 | 0 | 0 | 0 | 0 | 0 | 0 | -/doubtful | 0 |
| MMAS-8 | Jankowska-Polanska et al., 2014 ^197^ | 0 | ?/doubtful | ?/doubtful | +/very good | ^a^ | -/doubtful | 0 | 0 | -/very good | 0 |
| MMAS-8 | Kim et al., 2012 ^198^ | 0 | ?/doubtful | -/very good | -/inadequate | ^a^ | +/adequate | 0 | 0 | +/very good | -/very good |
| MMAS-8 | Korb-Savoldelli et al., 2013 ^199^ | 0 | +/doubtful | ?/very good | -/very good | ^a^ | -/very good | 0 | 0 | 0 | 0 |
| MMAS-8 | Lee et al., 2008 ^200^ | 0 | +/adequate | ?/adequate | -/very good | ^a^ | +/doubtful | 0 | 0 | +/very good | -/very good |
| MMAS-8 | Morisky et al., 2009 ^201^ | 0 | 0 | +/very good | +/very good | 0 | 0 | 0 | 0 | +/very good | -/very good |
| MMAS-8 | Nakhaeizadeh et al., 2019 ^202^ | 0 | +/doubtful | +/very good | +/inadequate | ^a^ | +/adequate | 0 | 0 | +/adequate | 0 |
| MMAS-8 | Sakthong et al., 2009 ^203^ | 0 | +/adequate | ?/adequate | -/inadequate | ^a^ | +/doubtful | 0 | 0 | +/very good | -/doubtful |
| MMAS-8 | Shilbayeh et al., 2018 ^204^ | 0 | 0 | 0 | -/adequate | 0 | 0 | 0 | 0 | -/adequate | -/very good |
| MMAS-8 | Tan et al., 2016 ^205^ | 0 | 0 | 0 | +/inadequate | 0 | +/adequate | 0 | 0 | -/inadequate | 0 |
| MMAS-8 | Wang et al., 2012 ^206^ | 0 | 0 | +/very good | +/very good | 0 | 0 | 0 | 0 | -/doubtful | 0 |
| MMAS-8 | Zongo et al., 2016 ^119^ | 0 | 0 | 0 | 0 | 0 | 0 | 0 | 0 | -/adequate | -/adequate |
| MMAS-8 | Zongo et al., 2016 ^207^ | 0 | 0 | ?/adequate | -/inadequate | 0 | 0 | 0 | 0 | 0 | 0 |
| MMAS-8 | Muntner et al., 2011^51^ | 0 | 0 | 0 | 0 | 0 | -/ very good | 0 | 0 | 0 | 0 |
| MMAS-8 (MIAS) | Osborn et al., 2016 ^208^ | 0 | 0 | 0 | -/very good | 0 | 0 | 0 | 0 | +/adequate | 0 |
| MMAS-8 (MMAS-8D) | Arnet et al., 2015 ^209^ | 0 | +/doubtful | ?/doubtful | -/very good | ^a^ | 0 | 0 | 0 | -/adequate | 0 |
| MMAS-8 (MMAS-K) | Shin et al., 2013 ^210^ | 0 | +/doubtful | ?/adequate | +/very good | ^a^ | 0 | 0 | 0 | +/very good | ?/adequate |
| MMAS-8 (MMAS-U) | Okello et al., 2016 ^211^ | 0 | +/adequate | ?/adequate | -/very good | ^a^ | -/adequate | 0 | 0 | 0 | 0 |
| MMAS-8 (OS-MMAS-8) | Reynolds et al., 2014 ^212^ | 0 | 0 | -/very good | +/very good | 0 | +/adequate | 0 | 0 | +/very good | ?/doubtful |
| MMAS-8 (OS-MMAS) | Reynolds et al., 2012 ^213^ | 0 | 0 | +/very good | +/very good | 0 | +/very good | 0 | 0 | +/very good | 0 |
| MMAS-9 | Wickersham et al., 2018 ^214^ | 0 | 0 | +/very good | -/very good | 0 | ?/doubtful | 0 | 0 | -/adequate | 0 |
| MNPS | Athavale et al., 2017 ^215^ | doubtful | ?/doubtful | +/very good | +/inadequate | 0 | 0 | 0 | 0 | +/doubtful | -/inadequate |
| MOS General Adherence Scale | Jerant et al., 2008 ^187^ | 0 | 0 | 0 | +/inadequate | 0 | 0 | 0 | 0 | -/doubtful | 0 |
| MS-TAQ | Wicks et al., 2011 ^216^ | doubtful | ?/doubtful | 0 | -/very good | 0 | 0 | 0 | 0 | +/very good | 0 |
| OEOMA | Resnick et al., 2003 ^217^ | inadequate | 0 | -/very good | +/very good | 0 | 0 | 0 | 0 | ?/doubtful | 0 |
| OEOMA (OEOMA-C) | Qi et al., 2014 ^218^ | 0 | +/adequate | +/very good | +/very good | ^a^ | 0 | 0 | 0 | -/adequate | 0 |
| PEDIA scale | Almeida-Brasil et al., 2019 ^219^ | doubtful | +/doubtful | ?/adequate | ?/doubtful | ^a^ | -/doubtful | 0 | 0 | +/doubtful | 0 |
| Pictographic self-efficacy scale | Kalichman et al., 2005 ^220^ | inadequate | +/adequate | 0 | -/very good | 0 | -/inadequate | 0 | 0 | -/adequate | 0 |
| POP | Friemann et al., 2013 ^102^ | 0 | 0 | 0 | 0 | 0 | 0 | 0 | 0 | +/adequate | 0 |
| PPQ | Zschocke et al., 2014 ^221^ | doubtful | +/doubtful | 0 | +/doubtful | 0 | 0 | 0 | 0 | 0 | 0 |
| ProMAS | Kleppe et al., 2005 ^222^ | doubtful | ±/doubtful | -/very good | +/doubtful | 0 | 0 | 0 | 0 | 0 | 0 |
| PT/PP | Jerant et al., 2018 ^187^ | 0 | 0 | 0 | ?/inadequate | 0 | -/inadequate | 0 | 0 | -/doubtful | 0 |
| PT/PP | Arnsten et al., 2001 ^223^ | 0 | 0 | 0 | 0 | 0 | 0 | 0 | 0 | +/adequate | 0 |
| QATOP | Teixeira et al., 2007 ^224^ | adequate | +/adequate | 0 | 0 | 0 | 0 | 0 | 0 | ?/inadequate | 0 |
| Question of Interest (Qis) | Walewski et al., 2004 ^225^ | 0 | ?/doubtful | 0 | 0 | 0 | 0 | 0 | 0 | -/inadequate | -/inadequate |
| SCI | Lewin et al., 2009 ^226^ | 0 | 0 | 0 | +/inadequate | 0 | -/adequate | 0 | 0 | +/very good | -/adequate |
| SCI | Mumtaz et al., 2016 ^227^ | 0 | ?/doubtful | -/very good | +/very good | ^a^ | 0 | 0 | 0 | -/adequate | 0 |
| SCI-R | Jansà et al., 2013 ^228^ | 0 | +/adequate | ?/adequate | +/very good | ^a^ | ?/inadequate | 0 | 0 | +/inadequate | +/inadequate |
| SCRAT | Alhomoud et al., 2016 ^229^ | 0 | 0 | 0 | +/very good | 0 | 0 | 0 | 0 | +/very good | +/very good |
| SCRAT | Lubinga et al., 2011 ^230^ | 0 | 0 | 0 | +/doubtful | 0 | 0 | 0 | 0 | +/inadequate | +/adequate |
| SDSCA | Cohen et al., 2010 ^178^ | 0 | 0 | 0 | 0 | 0 | 0 | 0 | 0 | -/inadequate | 0 |
| SDSCA | Gonzalez et al., 2013 ^55^ | 0 | 0 | 0 | 0 | 0 | 0 | 0 | 0 | +/doubtful | 0 |
| SEAMS | Pedrosa et al., 2016 ^231^ | 0 | 0 | ?/adequate | +/very good | 0 | 0 | 0 | 0 | +/inadequate | 0 |
| SEAMS | Pedrosa et al., 2016 ^232^ | 0 | +/adequate | 0 | +/very good | ^a^ | +/very good | 0 | 0 | +/very good | 0 |
| SEAMS | Risser et al., 2017 ^233^ | adequate | +/adequate | ?/adequate | +/very good | 0 | ?/doubtful | 0 | 0 | -/adequate | 0 |
| Self-efficacy scale | Najimi et al., 2017 ^234^ | inadequate | ?/adequate | ?/adequate | +/very good | 0 | 0 | 0 | 0 | ?/inadequate | 0 |
| Self-report measures of adherence | Blumberg et al., 2005 ^235^ | 0 | 0 | 0 | 0 | 0 | ?/adequate | 0 | 0 | +/adequate | 0 |
| Self-report on adherence | Prado et al., 2007 ^186^ | 0 | 0 | 0 | 0 | 0 | 0 | 0 | 0 | -/doubtful | +/doubtful |
| SEOMA (SEOMA-C) | Qi et al., 2014 ^218^ | 0 | +/adequate | -/very good | +/very good | ^a^ | 0 | 0 | 0 | +/adequate | 0 |
| SEOMA | Resnick et al., 2003 ^217^ | inadequate | 0 | -/very good | +/very good | 0 | 0 | 0 | 0 | ?/doubtful | +/doubtful |
| SERAD questionnaire | Munoz-Moreno, et al., 2007 ^236^ | 0 | ?/inadequate | 0 | 0 | 0 | 0 | 0 | 0 | ?/inadequate | 0 |
| SICT | Rofail et al., 2009 ^237^ | adequate | +/adequate | ?/adequate | +/very good | 0 | 0 | 0 | 0 | +/very good | 0 |
| *Sidorkiewicz et al*^c^ | Sidorkiewicz et al., 2016 ^238^ | adequate | +/adequate | 0 | 0 | 0 | -/adequate | 0 | 0 | +/doubtful | 0 |
| SMAQ | Knobel et al., 2002 ^239^ | inadequate | ?/doubtful | 0 | +/doubtful | 0 | +/very good | 0 | 0 | +/adequate | 0 |
| SMAQ | Ortega et al., 2011 ^240^ | 0 | 0 | 0 | 0 | 0 | +/doubtful | 0 | 0 | +/adequate | -/very good |
| SOC Questionnaire | Rathbun et al., 2007 ^241^ | 0 | 0 | 0 | 0 | 0 | 0 | 0 | 0 | ?/inadequate | 0 |
| SOC Questionnaire | Willey et al., 2000 ^242^ | inadequate | 0 | 0 | 0 | 0 | 0 | 0 | 0 | +/adequate | 0 |
| SPNS adherence survey | Julian et al., 2010 ^243^ | 0 | 0 | 0 | +/inadequate | 0 | 0 | 0 | 0 | +/adequate | 0 |
| SRSI^b^ | Buscher et al., 2011 ^54^ | 0 | 0 | 0 | 0 | 0 | 0 | 0 | 0 | +/adequate | 0 |
| SRSI^b^ | Feldman et al., 2013 ^244^ | 0 | 0 | 0 | 0 | 0 | 0 | 0 | 0 | +/inadequate | +/doubtful |
| SRSI^b^ | Gonzalez et al., 2013 ^55^ | 0 | 0 | 0 | 0 | 0 | 0 | 0 | 0 | +/doubtful | 0 |
| SRSI | Lu et al., 2008 ^245^ | 0 | 0 | 0 | 0 | 0 | 0 | 0 | 0 | -/inadequate | 0 |
| TAI | Gutiérrez-Pereyra et al., 2015 ^246^ | adequate | ?/adequate | ?/adequate | +/inadequate | 0 | +/very good | 0 | 0 | -/doubtful | -/very good |
| *Tan et al*^c^ | Tan et al., 2019 ^247^ | doubtful | ?/doubtful | ?/adequate | +/very good | 0 | +/very good | 0 | 0 | +/adequate | 0 |
| TAS-P | Mancebo et al., 2008 ^248^ | inadequate | 0 | 0 | 0 | 0 | +/very good | 0 | 0 | -/adequate | 0 |
| TASHP | He et al., 2016 ^249^ | 0 | 0 | +/very good | +/very good | 0 | 0 | 0 | 0 | +/adequate | -/adequate |
| TTAQ | Zschocke et al., 2015 ^221^ | doubtful | +/doubtful | 0 | +/doubtful | 0 | 0 | 0 | 0 | 0 | 0 |
| *Turcu-știolică et al*^c^ | Turcu-știolică et al., ^250^ | doubtful | +/doubtful | 0 | -/inadequate | 0 | 0 | 0 | 0 | -/inadequate | 0 |
| VAS | Buscher et al., 2018 ^54^ | 0 | 0 | 0 | 0 | 0 | 0 | 0 | 0 | +/adequate | 0 |
| VAS | Kalichman et al., 2011 ^56^ | 0 | 0 | 0 | 0 | 0 | -/inadequate | 0 | 0 | +/adequate | 0 |
| VAS | Kerr et al., 2009 ^86^ | 0 | 0 | 0 | 0 | 0 | 0 | 0 | 0 | +/adequate | 0 |
| VAS | Giordano et al., 2012 ^251^ | 0 | 0 | 0 | 0 | 0 | 0 | 0 | 0 | +/inadequate | 0 |
| VERITAS-PRN | Duncan et al., 2010 ^252^ | inadequate | ?//doubtful | 0 | +/very good | 0 | +/very good | 0 | 0 | +/inadequate | 0 |
| VERITAS-Pro | Duncan et al., 2010 ^253^ | adequate | +/adequate | 0 | +/very good | 0 | ?/doubtful | 0 | 0 | +/adequate | 0 |
| *Voils et al* ^c^ | Liau et al., 2019 ^254^ | 0 | +/very good | +/very good | -/very good | ^a^ | -/very good | 0 | 0 | +/very good | 0 |
| *Voils et al* ^c^ | Voils et al., 2012 ^15^ | doubtful | +/doubtful | +/very good | +/very good | 0 | -/adequate | 0 | 0 | -/very good | 0 |
| *Voils et al* ^c^(extent of nonadherence) | Blalock et al., 2019 ^255^ | 0 | 0 | +/very good | +/very good | 0 | 0 | 0 | 0 | +/doubtful | +/adequate |
| *Voils et al* ^c^(extent of nonadherence) | Cornelius et al., 2019 ^256^ | 0 | ?/doubtful | 0 | 0 | ^a^ | 0 | 0 | 0 | +/adequate | -/adequate |
| *Vreeman et al*^c^ | Vreeman et al., 2019 ^257^ | 0 | 0 | 0 | 0 | 0 | 0 | 0 | 0 | -/doubtful | -/doubtful |
| *Vreeman et al*^c^ | Vreeman et al., 2014 ^258^ | very good | ?/very good | 0 | 0 | ^a^ | 0 | 0 | 0 | 0 | 0 |
| Web-Ad-Q Questionnaire | Vale et al., 2018 ^259^ | doubtful | +/doubtful | 0 | 0 | 0 | +/inadequate | 0 | 0 | +/doubtful | 0 |
| *Wilson et al*^c^ | Phillips et al., 2017 ^260^ | 0 | 0 | 0 | +/very good | ^a^ | 0 | 0 | 0 | 0 | -/adequate |
| *Wilson et al*^c^ | Wilson et al., 2014^261^ | adequate | +/adequate | 0 | +/inadequate | 0 | 0 | 0 | 0 | 0 | 0 |
| *Wilson et al*^c^ | Wilson et al., 2016 ^262^ | 0 | 0 | 0 | +/inadequate | 0 | 0 | 0 | 0 | +/inadequate | 0 |

^a^ Only translation was done. Cross-cultural validation was not the aim of the study

^b^ Study assessed selected item domain to assess its measurement property

^c^ PROMs without proper names are labelled based on the last name of the first author who developed the instrument

**Legends**

+: Positive; -: Negative; ?: Indeterminate

0: Measurement property was not assessed by the study

**Abbreviations:**

AAAQ: Adult Asthma Adherence Questionnaire; AACTG: Adult AIDS Clinical Trials Group; AAS: Antidepressant Adherence Scale; ABQ: Adherence Barrier Questionnaire; ADEOS: Adherence Evaluation of Osteoporosis Treatment Questionnaire; AMBAS: Antipsychotic Medication Beliefs and Attitudes Scale; APRQ: Adherence to Pulmonary Rehabilitation Questionnaire; ARMS: Adherence to Refills and Medications Scale; ASK: Adherence Starts with Knowledge questionnaire; ASRQ: Adherence self-report questionnaire; BAASIS: Basel Assessment of Adherence to Immunosuppressive Medications Scale; BEMIB: Brief Evaluation of Medication Influences and Beliefs; BERMA: Beliefs Related to Medication Adherence; BMAS: Brief Medication Adherence Scale; BMCS: Beliefs about Medication Compliance Scale; BMQ: Brief Medication Questionnaire; CASE: Center for Adherence Support Evaluation; CDCI: Chronic Disease Compliance Instrument; CEAT-VIH: *Cuestionario para la Evaluacio´ n de la Adhesio´ n al Tratamiento Antirretroviral en Personas con Infeccio´ n por VIH y Sida’’*;CoSMO: Cohort Study of Medication Adherence Among Older Adults; CQR: Compliance Questionnaire on Rheumatology; DAI: Drug Attitude Inventory; DAMS: Diagnostic Adherence to Medication Scale; DMQ: Diabetes Management Questionnaire; DMSS: Diabetes Medication Self-efficacy Scale; DRAW: Drug Adherence Work-Up Tool; EBAS: Environmental Barriers to Adherence Scale; EDSQ: Eye-Drop Satisfaction Questionnaire; ESRD-AQ: End-Stage Renal Disease Adherence Questionnaire; GMAS: General Medicine Adherence Scale; GTCAT: Glaucoma Treatment Compliance Assessment Tool; HIV-IM: HIV Intention Measure; HIV-SQUAD: HIV Symptom Quality of Life Adherence Questionnaire; IADMAS: Iraqi Anti-Diabetic Medication Adherence Scale; IRT: Item Response Theory; ITAS: Immunosuppressant Therapy Adherence Scale; ITBS: Immunosuppressant Therapy Barrier Scale; Lasso- Least absolute shrinkage and selection operator; MAQ: Medication Adherence Questionnaire; MAR-Scale: Medication Adherence Reasons Scale MARS: Medication Adherence Rating Scale; MARS-5/9/10: 5/9/10-item Medication Adherence Report Scale; MASES: Medication Adherence Self-efficacy Scale; MASRI: Medication Adherence Self-Report Inventory; MEDS: Medication Adherence Estimation and Differentiation Scale; MIS-A: Medication Intake Survey-Asthma; MMAS: Morisky Medication Adherence Scale; MNPS: Medication Nonpersistence Scale; MOS: Medical Outcomes Study; MS-TAQ: Multiple Sclerosis Treatment Adherence Questionnaire; OEOMA: Outcome Expectations for Osteoporosis Medication Adherence Scale; PEDIA: Perceived Barriers to Antiretroviral Therapy Adherence Scale; POP: Patient Rating of Compliance Scale; PPQ: Patient Preference Questionnaire; ProMAS: Probabilistic Medication Adherence Scale: PT/PP: No. of pills taken/prescribed; QATOP: Questionnaire for Adherence with Topical Treatments in Psoriasis; SCI: Self-Care Inventory; SCRAT: Strathclyde Compliance Risk Assessment Tool; SDSCA: Summary of Diabetes Self-care Activities; SEAMS: Self-Efficacy for Appropriate Medication Adherence Scale; SEOMA: Self-Efficacy for Osteoporosis Medication Adherence Scale; SERAD: Self-Reported Adherence questionnaire; SICT: Satisfaction with iron chelation therapy; SMAQ: Simplified Medication Adherence Questionnaire; SOC: Stages of change model; SPNS: Special Projects of National Significance adherence tool; SRSI: Self-rating scale item; TAI: Test of the Adherence to Inhalers; TAS-P: Treatment Adherence Survey-Patient Version; TASHP: Therapeutic Adherence Scale for Hypertensive Patients; TTAQ: Topical Therapy Adherence Questionnaire; VAS: Visual analogue scale; VERITAS-PRN: Validated Hemophilia Regimen Treatment Adherence Scale – On-Demand; VERITAS-Pro: Validated Hemophilia Regimen Treatment Regimen Treatment Adherence Scale-Prophylaxis
